# Opioid Tapering and Opioid Overdose, Opioid Use Disorder, and Mortality Among Older Adults: A Nested Case–Control Study

**DOI:** 10.1007/s11606-025-09492-9

**Published:** 2025-04-22

**Authors:** Yi Yang, Prachi Prajapati, Sujith Ramachandran, Kaustuv Bhattacharya, Shadi Bazzazzadehgan, Shishir Maharjan, Ike Eriator, John P. Bentley

**Affiliations:** 1 Department of Pharmacy Administration, University of Mississippi School of Pharmacy, University, MS USA; 2 Center for Pharmaceutical Marketing and Management, University of Mississippi School of Pharmacy, University, MS USA; 3 Department of Anesthesiology, School of Medicine, University of Mississippi Medical Center, Jackson, MS USA

**Keywords:** opioid tapering, overdose, opioid use disorder, mortality, older adults

## Abstract

**Background:**

Opioid tapering has increased in recent years; however, evidence regarding its safety profile is lacking.

**Objective:**

To examine the relationships between opioid tapering and subsequent overdose (OD), opioid use disorder (OUD), and all-cause mortality among older adults on long-term opioid therapy (LTOT).

**Design:**

Nested case–control design.

**Participants:**

A cohort of older (≥ 65 years) Medicare beneficiaries with chronic non-cancer pain who were on LTOT was identified from 2012–2020 5% national Medicare claims data.

**Main Measures:**

The key independent variable was rate of opioid tapering, operationalized as a monthly dose change percentage with four levels: steady dose (± 10% dose change), slow tapering (10–40% dose reduction), rapid tapering (> 40% dose reduction), and dose escalation (> 10% dose increase). The outcome variables were OD, OUD, and all-cause mortality. Conditional logistic regression was conducted on matched samples to evaluate the associations between opioid tapering and the outcomes.

**Key Results:**

Among a cohort of 82,295, 1333 cases of OD, 4933 cases of OUD, and 5971 cases of all-cause mortality were identified. In primary analyses, after controlling for all covariates, compared with steady dose, the odds of OD were significantly lower (aOR = 0.74; 95% CI = 0.55–0.99) for rapid tapering and significantly higher (aOR = 2.08; 95% CI = 1.64–2.65) for dose escalation. Compared to steady dose, the odds of OUD were significantly lower (aOR = 0.53; 95% CI = 0.46–0.60) for rapid tapering and significantly higher (aOR = 1.60; 95% CI = 1.42–1.81) for dose escalation. Compared to steady dose, significantly higher odds for all-cause mortality were found among patients undergoing rapid tapering (aOR = 1.28; 95% CI = 1.14–1.44), and dose escalation (aOR = 1.51; 95% CI = 1.34–1.71). Sensitivity analyses showed that mortality outcome is sensitive to variations in cohort selections.

**Conclusion:**

The results suggest that any opioid dose change for patients on LTOT may negatively affect all-cause mortality. Clinicians should regularly assess patients on LTOT, considering the benefits and risks of treatment that incorporate evolving evidence on dose changes.

**Supplementary Information:**

The online version contains supplementary material available at 10.1007/s11606-025-09492-9.

## INTRODUCTION

The total number of opioid prescriptions and the number of older adults receiving long-term opioid therapy (LTOT) for chronic non-cancer pain (CNCP) in the USA has been declining in recent years.^1–3^ From 2011 to 2020, the total opioid prescriptions in the USA have dropped by almost 60%.^4^ This decrease in utilization is concordant with an increase in opioid tapering^5^ and discontinuation^6,7^ following the release of the Centers for Disease Control and Prevention (CDC) guideline for prescribing opioids for chronic pain in 2016.^8–12^ After the 2016 CDC guideline, there was a significant and immediate increase in opioid tapering, with approximately 52.6% of patients on high-dose LTOT experiencing dose reductions or discontinuation.^5,7,13^ However, despite the decline in opioid prescribing and increasing trend of opioid tapering, opioid-related morbidity, and mortality have not shown a parallel decline.^3,14–17^ While this may be partly explained by individuals moving from misusing prescription opioids to using illicit drugs, it is likely that some of the outcomes may be associated with opioid dose changes.^18,19^

Evidence regarding the relationship between changes in opioid dosage and outcomes is inconsistent. While Hallvik et al. reported that opioid discontinuation was associated with decreased risk of overdose, others found that opioid tapering was associated with increased risk of overdose but not associated with mortality.^20–23^ Recently, there has been an increased focus on the rate of opioid dose changes in opioid-related outcomes research. Research has shown that more rapid opioid tapering or lesser time to discontinuation was associated with increased risks of mental health crisis,^21,24^ overdose, suicide,^21,25,26^ opioid-related emergency department visits,^26^ and hospitalizations.^27^

Although LTOT is often prescribed for older adults to manage CNCP, these patients often have declining metabolic functions, polypharmacy, and multiple comorbidities, making them more likely to have greater susceptibility to adverse outcomes associated with LTOT.^28–31^ A significant proportion of older adults with chronic pain experience dose tapering to reduce or discontinue opioids.^5,6,32^ However, there is a lack of evidence regarding the safety and effectiveness of rate of opioid dose changes in older adults. In this study, we sought to investigate the relationship between opioid tapering and subsequent overdose (OD), opioid use disorder (OUD), and all-cause mortality among Medicare-enrolled older adults with CNCP who are receiving LTOT.

## METHODS

### Study Design and Data Source

This is a nested case–control study using the 2012–2020 5% national sample of Medicare claims data. Medicare inpatient, outpatient, and prescription drug data, along with beneficiary demographics, enrollment, diagnosis, and procedure codes, were linked by an encrypted beneficiary identifier. Data used in this study were made available under DUA #RSCH- 2023–59140 from the Centers for Medicare and Medicaid Services. This study was approved by the University of Mississippi Institutional Review Board (Protocol #21x- 245) and followed the Strengthening the Reporting of Observational Studies in Epidemiology (STROBE) guideline.

### Study Cohort

Medicare beneficiaries with CNCP were eligible for this study if they were on LTOT between July 1, 2012, and December 31, 2020. Based on previous literature, LTOT was defined as having at least three opioid prescription claims summing to a cumulative 45-day supply within any 90-day period during the study timeline.^1,24,33^ The “cohort entry date” was defined as the 91 st day after LTOT initiation. Other inclusion criteria were having to be at least 65 years of age or older at cohort entry date, having at least two claims with diagnoses for CNCP conditions (Appendix Table 1) in a 30-day period within the 6 months prior to cohort entry date (baseline period), and continuous enrollment in Medicare parts A, B, and D in the baseline period. Beneficiaries with a diagnosis of cancer (Appendix Table 2), those who were enrolled in hospice care within the baseline period, or those who were enrolled in a Medicare Advantage plan during the baseline period were excluded. Beneficiaries remained in the cohort until they had the outcomes of interest, diagnosis of cancer, loss of Medicare enrollment, enrollment in Medicare Advantage, or end of the study period, whichever came first. CDC guidance on calculating the total daily dose of opioids was followed to compute daily opioid dose, which was measured in morphine milligram equivalents (MME).^34^

### Selection of Cases and Controls

Individuals who experienced OD, OUD, or all-cause mortality were defined as cases if they had at least 7 days of opioids within the baseline hazard period (H_0_ or 120 to 90 days prior to the date of the outcome). The date of first occurrence of the outcome was defined as the index date for the cases. Medicare master beneficiary summary file was used to identify all-cause mortality. OUD was identified based on claims for any outpatient, emergency department (ED), physician office visit, or hospitalization with an International Classification of Diseases 9 th (ICD- 9) or 10 th revision (ICD- 10) diagnosis code for OUD (Appendix Table 3).^35^ OD cases were identified consistent with previous literature based on the presence of (1) any ICD- 9 or ICD- 10 code for opioid-related poisoning (Appendix Table 3); or (2) any ICD- 9 or ICD- 10 code for an opioid-related adverse event (Appendix Table 3) in addition to an ICD- 9 or ICD- 10 code for opioid overdose on the same day as the opioid-related adverse event.^30,33^

**Figure 1:**
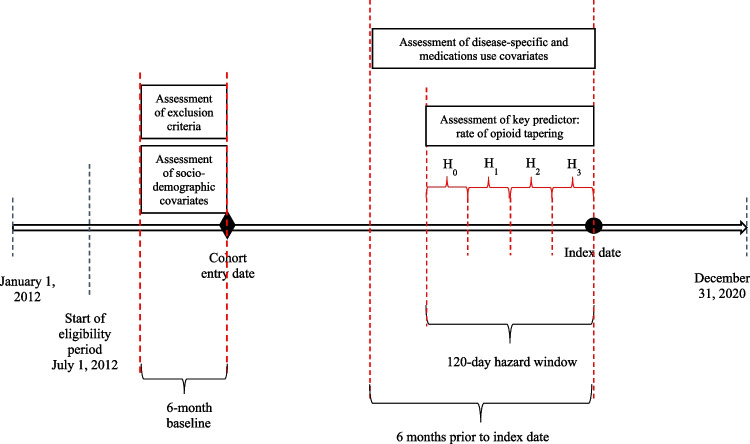
Study design diagram. Chronic non-cancer patients on long-term opioid therapy were followed until the first occurrence of the outcome (index date) from the start of the eligibility period (July 1, 2012). In the 120-day hazard window before the index date, the key independent variable, opioid tapering rate, was measured. While sociodemographic covariates were measured in the 6 months before cohort entry date, disease-specific and medications use were measured 6 months before the index date. H_0_, baseline hazard period—120 to 90 days before the index date; H_1_, first hazard period—90 to 60 days before the index date; H_2_, second hazard period—60 to 30 days before the index date; H_3_, third hazard period—30 days before the index date.

Controls were defined as individuals who did not experience the outcome of interest during the identification period. Incidence density sampling was used to identify controls. This sampling technique randomly samples individuals from all eligible controls as long as they have an equal or greater time at risk of an event than the corresponding matched case. This allows for each individual to serve as a control for more than one case and for controls to serve as a future case. Cases to controls were 1:2 on age (± 1 year) and time of cohort entry (± 30 days). Controls were assigned the index date of their matched cases. Like cases, controls were also required to have at least 7 days of opioid supply within the baseline hazard period (H_0_). Figure 1 presents the study design diagram.

### Key Exposure Variable: Rate of Opioid Tapering

The rate of opioid tapering was measured during the 120-day hazard window before the index date by dividing the window into four separate periods using this formula, $V=100*\left\{1-\text{exp}\left[\frac{\text{ln}\left(\frac{T+5}{B+5}\right)}{D}\right]\right\}$.^3,15^ In the formula, *B* is the average hazard baseline dose, *T* is the average tapered daily dose, and *D* is the time in months from the most recent month at the baseline dose (H_0_) to the earliest month at the tapered dose (minimum dose from H_1_ to H_3_, Fig. 1). The value of *T* was assigned as zero for beneficiaries who did not have any opioid fills in the 3rd hazard period (H_3_). A monthly percentage of dose reduction was obtained, such that higher values indicated more rapid tapering. Dose escalation was also captured in this formula since it assesses change in average monthly dose. However, dose escalation was represented by negative values of tapering rate. In this study, rate of opioid dose changes was categorized as a four-level variable: steady dose, defined as a monthly rate of opioid tapering less than 10% and greater than − 10%; slow tapering, a tapering rate between 10 and 40%; rapid tapering, a dose reduction rate greater than 40%; and dose escalation, a tapering rate lower than − 10%.

### Covariates

Sociodemographic variables including age, sex, race and ethnicity, and low-income subsidy status were measured in the baseline period. Disease-specific covariates and the Deyo-Charlson Comorbidity index (CCI) were measured in the 6 months prior to index date (Table 1).^36^ Beneficiaries were identified as having other medications use if, during the baseline hazard window, they had at least 90 days of supply of the following adjuvant analgesic medications: anticonvulsants, antidepressants, antipsychotics, benzodiazepines, non-benzodiazepine hypnotics, and skeletal muscle relaxants. Furthermore, an average opioid daily dose during baseline hazard window was computed.

**Table 1:** Description of Patient Demographic and Clinical Characteristics (*N*, %) by Outcome Measures

Patient characteristics	Full cohort (*N* = 89,295)	Opioid overdose			Opioid use disorder			All-cause mortality		
		Case (*N* = 1333)	Control* (*N* = 2666)	*p*-value	Case (*N* = 4933)	Control* (*N* = 9866)	*p*-value	Case (*N* = 5971)	Control* (*N* = 11,942)	*p*-value
Age mean (SD)	75.64 (8.1)	73.92 (7.1)	73.91 (7.1)		72.87 (6.6)	72.87 (6.6)		79.24 (8.9)	79.18 (8.9)	
Sex				0.49			0.897			< 0.001
Female	64,563 (72.3)	965 (72.4)	1957 (73.4)		3555 (72.1)	7120 (72.2)		4248 (71.1)	9411 (78.8)	
Race and ethnicity				< 0.001			< 0.001			0.005
Non-Hispanic White	71,443 (80.0)	1172 (87.9)	2233 (83.8)		4055 (82.2)	7848 (79.6)		4939 (82.7)	9839 (82.4)	
Black	9437 (10.6)	99 (7.4)	296 (11.1)		467 (9.5)	1094 (11.1)		599 (10.1)	1099 (9.2)	
Hispanic	5378 (6.0)	15 (1.1)	54 (2.0)		259 (5.2)	625 (6.3)		293 (4.9)	629 (5.3)	
Other racial groups	3037 (3.4)	47 (3.5)	83 (3.1)		152 (3.1)	299 (3.0)		140 (2.3)	375 (3.1)	
LIS enrollment				0.311			0.060			< 0.001
Yes	35,428 (39.7)	576 (43.2)	1197 (44.9)		2164 (43.9)	4489 (45.5)		3477 (58.2)	5129 (42.9)	
Multiple CNCP^†^	57,649 (64.6)	993 (74.5)	1419 (53.2)	< 0.001	3567 (72.3)	5807 (58.9)	< 0.001	3361 (56.3)	6081 (50.9)	< 0.001
Renal impairment^†^	10,780 (12.1)	215 (16.1)	272 (10.2)	< 0.001	620 (12.6)	1234 (12.5)	0.916	1920 (32.2)	1393 (11.7)	< 0.001
Hepatic impairment^†^	1462 (1.6)	17 (1.3)	25 (0.9)	0.324	85 (1.7)	140 (1.4)	0.154	206 (3.4)	105 (0.9)	< 0.001
Substance use^†^	21,913 (24.5)	518 (38.9)	663 (24.9)	< 0.001	1801 (36.5)	2922 (29.6)	< 0.001	2252 (37.7)	2355 (19.7)	< 0.001
Sleep disorders^†^	18,259 (20.4)	394 (29.6)	482 (18.0)	< 0.001	1226 (24.8)	2180 (22.1)	< 0.001	2471 (41.4)	1910 (16.0)	< 0.001
Parkinson^†^	1998 (2.2)	33 (2.5)	47 (1.7)	0.129	114 (2.3)	211 (2.1)	0.500	281 (4.7)	276 (2.3)	< 0.001
Mental disorder^†^	11,546 (12.9)	156 (11.7)	177 (6.6)	< 0.001	658 (13.3)	1031 (10.4)	< 0.001	1011 (16.9)	1131 (9.5)	< 0.001
COPD^†^	13,698 (15.3)	95 (7.1)	72 (2.7)	< 0.001	723 (14.7)	1289 (13.1)	0.008	1385 (23.2)	1269 (10.6)	< 0.001
Concomitant medication use^§^	NA	929 (69.7)	1624 (40.9)		3286 (66.6)	6106 (61.9)	< 0.001	3699 (61.9)	6721 (56.3)	< 0.001
CCI^†^				< 0.001			0.053			< 0.001
0	52,012 (58.2)	204 (15.3)	638 (23.9)		2496 (50.6)	5141 (52.1)		1627 (27.2)	6822 (57.1)	
1 to 2	30,446 (34.1)	447 (33.5)	1023 (38.4)		1835 (37.2)	3471 (35.2)		2630 (44.1)	4127 (34.6)	
≥ 3	6837 (7.7)	682 (51.2)	1005 (37.7)		602 (12.2)	1254 (12.7)		1714 (28.7)	993 (8.3)	
Mean opioid daily dose during baseline hazard period^‡^				< 0.001			< 0.001			< 0.001
< 20	NA	213 (16.0)	777 (29.1)		868 (17.6)	2842 (28.8)		1953 (32.7)	4228 (35.4)	
20–50	NA	543 (40.8)	1188 (44.6)		2084 (42.2)	4336 (43.9)		2530 (42.4)	5111 (42.8)	
≥ 50	NA	577 (43.4)	701 (26.3)		1981 (40.2)	2688 (27.3)		1488 (24.9)	2603 (21.8)	
Opioid tapering^﻿§^				< 0.001			< 0.001			< 0.001
Steady dose	NA	937 (70.3)	2030 (76.1)		3586 (72.7)	7130 (72.3)		3739 (62.6)	8534 (71.5)	
Slow tapering	NA	111 (8.3)	206 (7.7)		448 (9.1)	796 (8.1)		617 (10.4)	1114 (9.3)	
Rapid tapering	NA	86 (6.5)	225 (8.4)		318 (6.4)	1168 (11.8)		921 (15.4)	1304 (10.9)	
Dose escalation	NA	199 (14.9)	205 (7.7)		581 (11.8)	772 (7.8)		694 (11.6)	990 (8.3)	

*Abbreviations*: *LIS*, low income status; *COPD*, chronic obstructive pulmonary disease; *CCI*, Deyo-Charlson Comorbidity Index; *CNCP*, chronic non-cancer pain; *MME*, morphine milligram equivalents

^*^Cases and controls were matched based on age (± 1 year) and time of cohort entry (± 30 days)

^†^The variable was assessed during 6 months prior to index date

^‡^The variable was assessed during baseline hazard window, which is 120 to 90 days before the index date

^§^The variable was assessed during hazard period, which is 120 days prior to index date

### Sensitivity Analyses

Four sensitivity analyses were conducted to assess the robustness of the primary analysis. These included replications of the primary analysis after restricting the study population to substance use-naïve individuals (Appendix 5), those initiating (incident) LTOT (Appendix 6), individuals on LTOT for at least one year (Appendix 7), and requiring cases and controls to have at least 14 days of opioid use during the baseline hazard period (Appendix 8).

### Statistical Analysis

Baseline characteristics of the study cohort were summarized for all study variables. McNemar’s test or Cochran-Mantel–Haenszel test was used to test differences in baseline characteristics between the matched cases and controls. Unadjusted and adjusted analyses were conducted to evaluate the associations between opioid tapering and the outcomes of interest using conditional logistic regression, accounting for the matching. Adjusted odds ratios (aORs) and 95% confidence intervals (CI) were reported. SAS version 9.4 (Cary, NC) was used for data management and analyses.

## RESULTS

### Study Cohort

**Figure 2:**
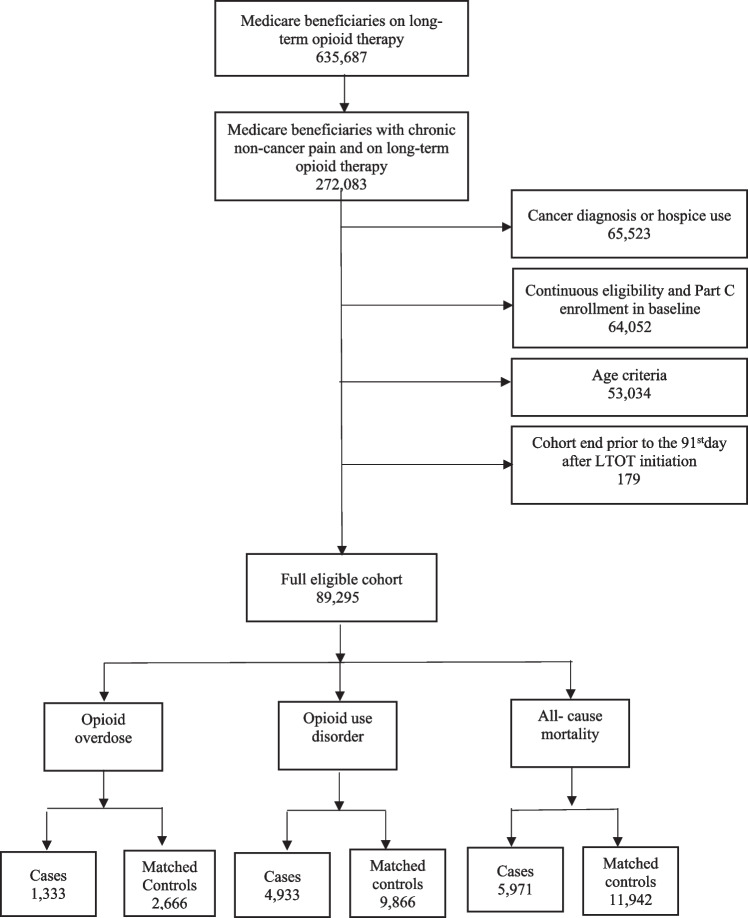
Flowchart for selection of cases and controls for overdose, opioid use disorder, and all-cause mortality. Among chronic non-cancer patients on long-term opioid therapy, those with cancer or hospice use, no continuous enrollment in Medicare parts A, B, and D, and any enrollment in Medicare part C during the 6 months before cohort entry date were excluded. Additionally, those with cohort end before cohort entry date were excluded as well. With these exclusions, the full eligible cohort was determined, and these beneficiaries were followed up for occurrence of outcomes (cases) and subsequently matched controls.

The final eligible cohort consisted of 89,295 Medicare beneficiaries (Fig. 2). The demographic and clinical characteristics of the full cohort and three sets of matched case–control pairs corresponding to each of the outcome measures (i.e., OD, OUD, and all-cause mortality) are presented in Table 1. The mean age of the full cohort was 75.64 years (standard deviation [SD] = 8.1).

As shown in Fig. 2, within the full cohort, we identified 1333 cases of OD, 4933 cases of OUD, and 5971 cases of all-cause mortality. For OD, OUD, and mortality cases, the median durations of time between cohort entry and the outcomes were 853 days (interquartile range [IQR]: 304–1460), 725.5 days (IQR: 273.5–1314), and 520 days (IQR: 177–1129), respectively.

### Results of Primary Analyses

The unadjusted results of the relationship between the rate of opioid dose change and OD, OUD, and mortality are shown in Table 2. Table 3 presents the adjusted relationships between rate of opioid dose changes and OD, OUD, and all-cause mortality. Compared to older adults on a steady dose, the odds of experiencing OD were significantly lower for those undergoing rapid tapering (aOR = 0.741; 95% CI: 0.553–0.992) and significantly higher for individuals experiencing dose escalation (aOR = 2.081; 95% CI: 1.636–2.646). The odds of experiencing OUD were slightly higher (while not statistically significant), among individuals undergoing slow tapering (aOR = 1.006; 95% CI: 0.883–1.145) compared to the individuals on a steady dose. Individuals undergoing rapid tapering had 47% (aOR = 0.527; 95% CI: 0.460–0.603) lower odds of experiencing OUD while dose escalation (aOR = 1.605; 95% CI: 1.421–1.814) was found to be significantly associated with the OUD, compared with a steady dose. The odds of all-cause mortality were significantly higher for individuals undergoing rapid tapering (aOR: 1.281; 95% CI: 1.141, 1.439) and dose escalation (aOR: 1.513; 95% CI: 1.336, 1.714) compared to those on a steady dose.

**Table 2:** Unadjusted Relationships Between Rate of Opioid Dose Changes and Opioid Overdose, Opioid Use Disorder, and All-Cause Mortality

Opioid dose changes	Opioid overdose		Opioid use disorder		All-cause mortality	
	Unadjusted odds ratio (95% CI)	*p*-value	Unadjusted odds ratio (95% CI)	*p*-value	Unadjusted odds ratio (95% CI)	*p*-value
Steady dose	Reference		Reference		Reference	
Slow tapering	1.144 (0.892–1.467)	0.290	1.118 (0.988–1.267)	0.078	1.284 (1.154–1.428)	< 0.001
Rapid tapering	0.840 (0.645–1.096)	0.199	0.541 (0.474–0.617)	< 0.001	1.636 (1.489–1.798)	< 0.001
Dose escalation	2.101 (1.698–2.598)	< 0.001	1.495 (1.331–1.680)	< 0.001	1.611 (1.450–1.790)	< 0.001

**Table 3:** Adjusted Relationships Between Rate of Opioid Dose Changes and Opioid Overdose, Opioid Use Disorder, and All-Cause Mortality

Patient characteristics	Opioid overdose		Opioid use disorder		All-cause mortality	
	Adjusted odds ratio (95% CI)	*p*-value	Adjusted odds ratio (95% CI)	*p*-value	Adjusted odds ratio (95% CI)	*p*-value
Rate of opioid dose changes						
Steady dose	Reference		Reference		Reference	
Slow tapering	0.966 (0.735–1.27)	0.804	1.006 (0.883–1.145)	0.934	1.092 (0.961–1.241)	0.175
Rapid tapering	0.741 (0.553–0.992)	0.044	0.527 (0.460–0.603)	< 0.001	1.281 (1.141–1.439)	< 0.001
Dose escalation	2.081 (1.636–2.646)	<.001	1.605 (1.421–1.814)	< 0.001	1.513 (1.336–1.714)	< 0.001
Sex						
Female	1.007 (0.85–1.194)	0.934	1.105 (0.935–1.103)	0.722	0.725 (0.663–0.793)	< 0.001
Race and ethnicity						
Non-Hispanic White	Reference		Reference		Reference	
Black	0.723 (0.552–0.946)	0.018	0.955 (0.842–1.084)	0.478	0.778 (0.682–0.888)	< 0.001
Hispanic	0.793 (0.426–1.474)	0.463	0.891 (0.758–1.046)	0.157	0.675 (0.567–0.803)	< 0.001
Other racial groups	1.511 (0.999–2.285)	0.051	1.105 (0.896–1.365)	0.351	0.672 (0.532–0.848)	< 0.001
LIS enrollment						
Yes	0.934 (0.797–1.095)	0.402	1.014 (0.938–1.097)	0.727	1.787 (1.651–1.934)	< 0.001
Multiple CNCP	2.013 (1.712–2.368)	< 0.001	1.686 (1.555–1.829)	< 0.001	0.856 (0.791–0.927)	< 0.001
Renal impairment	1.083 (0.859–1.365)	0.499	0.925 (0.825–1.036)	0.178	2.017 (1.834–2.218)	< 0.001
Hepatic impairment	0.804 (0.388–1.665)	0.556	1.135 (0.855–1.507)	0.380	2.059 (1.539–2.756)	< 0.001
Substance use	1.37 (1.16–1.618)	< 0.001	1.219 (1.124–1.322)	< 0.001	1.639 (1.499–1.792)	< 0.001
Sleep disorders	1.233 (1.031–1.476)	0.220	1.035 (0.946–1.132)	0.453	2.383 (2.181–2.604)	< 0.001
Parkinson	0.98 (0.59–1.628)	0.938	0.973 (0.764–1.239)	0.824	1.844 (1.509–2.254)	< 0.001
Mental disorder	1.49 (1.106–2.008)	0.009	1.160 (1.027–1.309)	0.017	1.503 (1.326–1.704)	< 0.001
COPD	1.477 (1.002–2.175)	0.049	1.016 (0.896–1.151)	0.810	1.373 (1.208–1.561)	< 0.001
Concomitant medication use	1.286 (1.094–1.512)	0.002	1.080 (0.999–1.169)	0.054	1.168 (1.080–1.264)	< 0.001
CCI						
0	Reference		Reference		Reference	
1 to 2	1.225 (0.994–1.511)	0.060	0.955 (0.877–1.039)	0.284	1.744 (1.595–1.906)	< 0.001
≥ 3	1.56 (1.252–1.944)	< 0.001	0.811 (0.714–0.922)	0.001	3.343 (2.962–3.774)	< 0.001
Average MME						
< 20	Reference		Reference		Reference	
20–50	1.787 (1.457–2.192)	< 0.001	1.686 (1.527–1.861)	< 0.001	1.035 (0.948–1.130)	0.444
≥ 50	3.534 (2.796–4.467)	< 0.001	2.753 (2.466–3.075)	< 0.001	1.234 (1.108–1.375)	< 0.001

*Abbreviations*: *LIS*, low-income status; *COPD*, chronic obstructive pulmonary disease; *CCI*, Deyo-Charlson Comorbidity Index; *CNCP*, chronic non-cancer pain; *MME*, morphine milligram equivalents

### Results of Sensitivity Analyses

Sensitivity analyses (Appendices 5–8) demonstrate that the primary study results on opioid-related outcomes (OD and OUD) are largely robust. However, risk of all-cause mortality resulting from slow tapering was found to be significantly elevated in variations of the study cohort (definition of LTOT and cases/controls).

## DISCUSSION

In this nested case–control study of older Medicare beneficiaries with CNCP who were receiving LTOT, we found that compared with a steady opioid dose, slow tapering (monthly dose reduction of 10–40%) was shown to not be significantly associated with increases in OD, OUD, and all-cause mortality. In contrast, rapid tapering with monthly dose reduction > 40% was associated with significantly increased risk of all-cause mortality but potentially had a protective effect against OD and OUD. Opioid dose escalation of > 10% per month was harmful in older adults with CNCP who were receiving LTOT as it was associated with increased risks for OD, OUD, and mortality.

LTOT is associated with increased risks of adverse health outcomes especially in older adults.^28–30,33,37^ As a result, many prescribers and even prescribing guidelines are considering opioid dose tapering as an approach to mitigate the harms attributable to LTOT.^38–40^ However, without evidence comparing the benefits and risks of different rates of tapering, well-intentioned reduction in opioid exposure can sometimes result in unintended, severe consequences.^21,24,41–44^ Our study systematically examined the potential adverse outcomes associated with rates of opioid dose change to provide much-needed real-world evidence on the safety of opioid dose changes in older adults. We found that slow tapering did not result in a reduction of risk of OD, or OUD. Conversely, slow tapering was associated with an increased risk of all-cause mortality in some populations examined in the sensitivity analysis. These results raise questions about the benefits of tapering from LTOT, even at slow rates, and demand closer re-examination of benefits and risks of any opioid dose change in patients receiving LTOT. For patients already receiving LTOT, if clinicians and patients agree that tapering is necessary, slow and gradual tapering, as recommended by the CDC, at a dose reduction of 10% per week as a starting point or as slow as 10% per month for long-term opioid users may help patients avoid withdrawal symptoms and opioid-related adverse events.^8,45^ The James et al. study found that discontinuation of chronic opioids was associated with an increased risk of mortality, but they did not examine different rates of opioid tapering.^22^ Our study adds to this evidence and shows that rapid tapering was significantly associated with mortality. Opioid tapering has also been reported to increase the risks of overdose, withdrawal symptoms, mental health crisis, and suicide, all of which can lead to mortality.^5,21,24,27,46^ The US Department of Health and Human Services and the CDC’s 2022 clinical practice guideline for prescribing opioids for pain recommend against rapid tapering or sudden discontinuation of opioids in patients on LTOT, as patients may experience an exacerbation of pain, psychological distress, suicidal thoughts, overdose death, or engage in illicit drug use.^9,45,47^ Hence, it is important for clinicians and patients to carefully weigh the benefits and risks of different tapering strategies with different rates of tapering; if tapering is warranted, it should be gradual and individualized.^8,43–45,48,49^ Sensitivity analysis of slow tapering in populations with incident and long-term LTOT showed a significant increase in risk of all-cause mortality, whereas the primary analysis did not such show such risks. This inconsistency likely reflects heterogeneity in severity of comorbidities in these cohorts, highlighting the downstream implications of opioid tapering among individuals with significant comorbidities. Further, the likelihood of individualized patient-centered tapering, which is important for the safety of the slow taper, may also be different across these cohorts. However, this study could not assess whether the tapering was conducted with appropriate patient involvement. Given the considerable variability in the definition of LTOT in the literature, future research should explore the impact of comorbidities and patient involvement in opioid tapering.

We observed a 26% reduction in the odds of OD and a 47% reduction in the odds of OUD associated with rapid tapering compared to steady dose. Although previous studies^21,25^ have suggested that rapid tapering increases the risk of OD, unlike these studies that primarily focused on younger individuals, our study focuses on older adults, who may be less likely to seek illicit drugs to compensate for opioid dose reductions. In comparison, patients undergoing slow tapering often have higher baseline risks, which may explain the lack of reductions in OD risk observed with this approach. Further, patients undergoing rapid tapering are more likely to discontinue opioids compared to those experiencing slow tapering, and the resulting reduction in risk of OD may be more apparent from a stoppage in opioid use than a reduction in dosage. The reduced odds of OUD finding can likely be explained by the definition of OUD itself. At least four of 11 diagnostic criteria for OUD outlined in the Diagnostic and Statistical Manual of Mental Disorders (DSM- 5) are in sharp contrast with the concept of opioid tapering and discontinuation.^50^ It is likely that individuals with unmanaged severe pain and those unable or unwilling to taper are more likely to have frequent interactions with the healthcare system. As a result, they are more likely to be labeled as having OUD, although this may not reflect an actual incidence of OUD itself.^51^

This study focused on a special population, older adults with CNCP who were receiving LTOT. Unlike their younger counterparts, the physiological changes of aging affect older patients’ ability to metabolize drugs. They are also more likely to have multiple comorbidities and need multiple medications, all of which make managing older patients on LTOT a challenge.^28–30^ For patients on LTOT, the benefits and risks of both LTOT and tapering should be carefully evaluated before initiating dose changes.^8,45^ Overall, slow opioid tapering is relatively safe and can be a viable approach to gradually reduce opioid doses, especially in patients where benefits of LTOT do not outweigh risks. In these situations, clinicians should work with patients to closely monitor any signs of adverse events. Rapid tapering should not be recommended as a tapering strategy as it is associated with significantly increased risks of mortality. In this study, monthly dose escalation of 10% or above has been associated with two times the odds of OD, 60.5% increase in odds of OUD and a 51.3% increase in odds of all-cause mortality among older CNCP adults on LTOT, suggesting that nontrivial dose escalation can increase risk of harm. In situations where pain is not well managed with LTOT, non-opioid therapies should be prioritized over opioid dose escalation.

### Limitations

Our study has several limitations. First, this study is observational and the associations between rates of opioid dose changes and adverse health outcomes may not be interpreted as causal. Second, administrative claims data are unable to identify use of illicit opioids or differentiate between whether prescriptions are used as needed or daily. However, recurring refill behavior or lack thereof can still indicate patterns of use of filled prescriptions, leading to reasonable assumptions in patterns of tapering.^45^ Additionally, despite accounting for history of substance use and conducting sensitivity analyses, the relationship between rates of opioid tapering and OUD may still be biased by confounding by indication as the concern that a patient may have OUD is the main reason for rapid tapering. The look-back period for identifying patients with substance abuse history/substance use disorder in sensitivity analysis was six months. However, there may be misclassification bias associated with this assessment since diagnosis codes for substance use related conditions are irregularly applied.^52,53^ Also, use of ICD diagnosis codes for identification of OD and OUD outcomes have shown limited accuracy and may lead to misclassification bias among outcomes.^54^ Relying on ICD codes to identify OD cases does not allow us to capture fatal overdoses occurring outside healthcare settings, which may contribute to the observed increases in all-cause mortality with rapid and slow tapering in some analyses. Future research should examine the accuracy of identification of OD and OUD and the relationship with tapering strategies. Furthermore, we used prescription drug fill data to identify LTOT and we were unable to verify whether patients actually consumed the opioids, although literature has shown that a filled prescription is a reliable proxy measure for actual medication use,^55^ but this relationship is untested for opioid medications.

## CONCLUSIONS

The results of this study suggest that among certain older CNCP patients who are on LTOT, slow tapering may be associated with increased risks of all-cause mortality. Rapid tapering was associated with significantly increased risk of all-cause mortality but had a potentially protective effect against OD and OUD. Opioid dose escalation may be harmful and was associated with increased risks for OD, OUD, and mortality. Clinicians should evaluate patients on LTOT to weigh the benefits and risks of treatment that incorporates evolving evidence on opioid dose changes and involving patients in the decision-making process.

## Supplementary Information

Below is the link to the electronic supplementary material.

Supplementary file1 (DOCX 91 KB)

## Data Availability

The data for this study was obtained via a data use agreement (DUA) with the Centers for Medicare and Medicaid Services (CMS) and cannot be shared publicly. However, data can be accessed, subject to approval and DUA, from the CMS (https://resdac.org/).
