# Supplementary material for: Opioid Tapering and Opioid Overdose, Opioid Use Disorder, and Mortality Among Older Adults: A Nested Case–Control Study
Source: J Gen Intern Med. 2025 Apr 22;40(12):2896–905. doi: 10.1007/s11606-025-09492-9 (PMC12463796; doi:10.1007/s11606-025-09492-9)
Supplement: Supplementary file 1 — Supplementary file1 (DOCX 91 KB) [file 11606_2025_9492_MOESM1_ESM.docx]

**Appendix Table 1: Diagnosis codes used to identify chronic non-cancer pain (CNCP)**

| **CNCP Condition** | **ICD-9-CM** | **ICD-10-CM** |
| --- | --- | --- |
| Rheumatoid Arthritis/Osteoarthritis | 714.0, 714.1, 714.2, 714.30, 714.31, 714.32, 714.33, 715.00, 715.04, 715.09, 715.10, 715.11, 715.12, 715.13, 715.14, 715.15, 715.16, 715.17, 715.18, 715.20, 715.21, 715.22, 715.23, 715.24, 715.25, 715.26, 715.27, 715.28, 715.30, 715.31, 715.32, 715.33, 715.34, 715.35, 715.36, 715.37, 715.38, 715.80, 715.89, 715.90, 715.91, 715.92, 715.93, 715.94, 715.95, 715.96, 715.97, 715.98, 720.0, 721.0, 721.1, 721.2, 721.3, 721.90, 721.91 | M05.00, M05.011, M05.012, M05.019, M05.021, M05.022, M05.029, M05.031, M05.032, M05.039, M05.041, M05.042, M05.049, M05.051, M05.052, M05.059, M05.061, M05.062, M05.069, M05.071, M05.072, M05.079, M05.09, M05.20, M05.211, M05.212, M05.219, M05.221, M05.222, M05.229, M05.231, M05.232, M05.239, M05.241, M05.242, M05.249, M05.251, M05.252, M05.259, M05.261, M05.262, M05.269, M05.271, M05.272, M05.279, M05.29, M05.30, M05.311, M05.312, M05.319, M05.321, M05.322, M05.329, M05.331, M05.332, M05.339, M05.341, M05.342, M05.349, M05.351, M05.352, M05.359, M05.361, M05.362, M05.369, M05.371, M05.372, M05.379, M05.39, M05.40, M05.411, M05.412, M05.419, M05.421, M05.422, M05.429, M05.431, M05.432, M05.439, M05.441, M05.442, M05.449, M05.451, M05.452, M05.459, M05.461, M05.462, M05.469, M05.471, M05.472, M05.479, M05.49, M05.50, M05.511, M05.512, M05.519, M05.521, M05.522, M05.529, M05.531, M05.532, M05.539, M05.541, M05.542, M05.549, M05.551, M05.552, M05.559, M05.561, M05.562, M05.569, M05.571, M05.572, M05.579, M05.59, M05.60, M05.611, M05.612, M05.619, M05.621, M05.622, M05.629, M05.631, M05.632, M05.639, M05.641, M05.642, M05.649, M05.651, M05.652, M05.659, M05.661, M05.662, M05.669, M05.671, M05.672, M05.679, M05.69, M05.70, M05.711, M05.712, M05.719, M05.721, M05.722, M05.729, M05.731, M05.732, M05.739, M05.741, M05.742, M05.749, M05.751, M05.752, M05.759, M05.761, M05.762, M05.769, M05.771, M05.772, M05.779, M05.79, M05.80, M05.811, M05.812, M05.819, M06.041, M06.042, M06.049, M06.051, M06.052, M06.059, M06.061, M06.062, M06.069, M06.071, M06.072, M06.079, M06.08, M06.09, M06.1, M06.20, M06.211, M06.212, M06.219, M06.221, M06.222, M06.229, M06.231, M06.232, M06.239, M06.241, M06.242, M06.249, M06.251, M06.252, M06.259, M06.261, M06.262, M06.269, M06.271, M06.272, M06.279, M06.28, M06.29, M06.30, M06.311, M06.312, M06.319, M06.321, M06.322, M06.329, M06.331, M06.332, M06.339, M06.341, M06.342, M06.349, M06.351, M06.352, M06.359, M06.361, M06.362, M06.369, M06.371, M06.372, M06.379, M06.38, M06.39, M06.80, M06.811, M06.812, M06.819, M06.821, M06.822, M06.829, M06.831, M06.832, M06.839, M06.841, M06.842, M06.849, M06.851, M06.852, M06.859, M06.861, M06.862, M06.869, M06.871, M06.872, M06.879, M06.88, M06.89, M06.9, M08.00, M08.011, M08.012, M08.019, M08.021, M08.022, M08.029, M08.031, M08.032, M08.039 |
| Back Pain | 724.5, 724.2, 724.00 | M54.5, M54.9, M54.89, M54.5, M54.50, M54.55, M54.56, M54.57, M54.58, M54.59, M54.6 |
| Extreme Pain | 338.1, 338.11, 338.12, 338.18, 338.19 | R52.0, R52.00 |
| Chest Pain | 786.5 | R07.9, R07, R07.1, R07.10, R07.3, R07.30, R07.4, R07.40 |
| Neuropathy | 356.8 | G60.8 |
| Temporomandibular Pain | 524.6 | M26.60, M26.69 |
| Neck Pain | 723.1 | M54.2 |
| Fibromyalgia | 729.1 | M79.7 |
| Diabetic Neuropathy | 250.6 | E11.40 |
| Radiculopathy | 724.4 | M54.14, M54.15, M54.16, M54.17 |
| Chronic Pancreatitis | 577.1 | K86.1 |
| Neuropathic Pain | 354.4, 355.71, 337.29, 355.9, 337.21, 337.22, 337.20, 338.28,053.19, 053.12, 053.10 | G90.50, G90.51, G90.52, G90.59, G58.9, G90.529, G90.519, G57.70, G56.40, B02.29, B02.22, G89.28 |

ICD-9-CM=The International Classification of Disease, 9th Revision, Clinical Modification;

ICD-10-CM=The International Classification of Disease, 10th Revision, Clinical Modification.

**Appendix Table 2. Diagnosis codes to identify cancer for exclusion criteria**

| **Cancer type** | **ICD-9-CM codes** | **ICD-10-CM codes** |
| --- | --- | --- |
| Malignant Neoplasm of Lip, Oral Cavity, And Pharynx | 140, 140.1, 140.3, 140.4, 140.5, 140.6, 140.8, 140.9, 141, 141.1, 141.2, 141.3, 141.4, 141.5, 141.6, 141.8, 141.9, 142, 142.1, 142.2, 142.8, 142.9, 143, 143.1, 143.8, 143.9, 144, 144.1, 144.8, 144.9, 145, 145.1, 145.2, 145.3, 145.4, 145.5, 145.6, 145.8, 145.9, 146, 146.1, 146.2, 146.3, 146.4, 146.5, 146.6, 146.7, 146.8, 146.9, 147, 147.1, 147.2, 147.3, 147.8, 147.9, 148, 148.1, 148.2, 148.3, 148.8, 148.9, 149, 149.1, 149.8, 149.9 | C00.0, C00.1, C00.2, C00.3, C00.4, C00.5, C00.6, C00.8, C00.9, C01, C02.0, C02.1, C02.2, C02.3, C02.4, C02.8, C02.9, C03.0, C03.1, C03.9, C04.0, C04.1, C04.8, C04.9, C05.0, C05.1, C05.2, C05.8, C05.9, C06.0, C06.1, C06.2, C06.80, C06.89, C06.9, C07, C08.0, C08.1, C08.9, C09.0, C09.1, C09.8, C09.9, C10.0, C10.1, C10.2, C10.3, C10.4, C10.8, C10.9, C11.0, C11.1, C11.2, C11.3, C11.8, C11.9, C12, C13.0, C13.1, C13.2, C13.8, C13.9, C14.0, C14.2, C14.8 |
| Malignant Neoplasm Of Digestive Organs And Peritoneum | 150, 150.1, 150.2, 150.3, 150.4, 150.5, 150.8, 150.9, 151, 151.1, 151.2, 151.3, 151.4, 151.5, 151.6, 151.8, 151.9, 152, 152.1, 152.2, 152.3, 152.8, 152.9, 153, 153.1, 153.2, 153.3, 153.4, 153.5, 153.6, 153.7, 153.8, 153.9, 154, 154.1, 154.2, 154.3, 154.8, 155, 155.1, 155.2, 156, 156.1, 156.2, 156.8, 156.9, 157, 157.1, 157.2, 157.3, 157.4, 157.8, 157.9, 158, 158.8, 158.9, 159, 159.1, 159.8, 159.9 | C15.3, C15.4, C15.5, C15.8, C15.9, C16.0, C16.1, C16.2, C16.3, C16.4, C16.5, C16.6, C16.8, C16.9, C17.0, C17.1, C17.2, C17.3, C17.8, C17.9, C18.0, C18.1, C18.2, C18.3, C18.4, C18.5, C18.6, C18.7, C18.8, C18.9, C19, C20, C21.0, C21.1, C21.2, C21.8, C22.0, C22.1, C22.2, C22.3, C22.4, C22.7, C22.8, C22.9, C23, C24.0, C24.1, C24.8, C24.9, C25.0, C25.1, C25.2, C25.3, C25.4, C25.7, C25.8, C25.9, C26.0, C26.1, C26.9 |
| Malignant Neoplasm Of Respiratory And Intrathoracic Organs | 160, 160.1, 160.2, 160.3, 160.4, 160.5, 160.8, 160.9, 161, 161.1, 161.2, 161.3, 161.8, 161.9, 162, 162.2, 162.3, 162.4, 162.5, 162.8, 162.9, 163, 163.1, 163.8, 163.9, 164, 164.1, 164.2, 164.3, 164.8, 164.9, 165, 165.8, 165.9 | C30.0, C30.1, C31.0, C31.1, C31.2, C31.3, C31.8, C31.9, C32.0, C32.1, C32.2, C32.3, C32.8, C32.9, C33, C34.00, C34.01, C34.02, C34.10, C34.11, C34.12, C34.2, C34.30, C34.31, C34.32, C34.80, C34.81, C34.82, C34.90, C34.91, C34.92, C37, C38.0, C38.1, C38.2, C38.3, C38.4, C38.8, C39.0, C39.9 |
| Malignant Neoplasm Of Bone, Connective Tissue, Skin, And Breast | 170, 170.1, 170.2, 170.3, 170.4, 170.5, 170.6, 170.7, 170.8, 170.9, 171, 171.2, 171.3, 171.4, 171.5, 171.6, 171.7, 171.8, 171.9, 172, 172.1, 172.2, 172.3, 172.4, 172.5, 172.6, 172.7, 172.8, 172.9, 174, 174.1, 174.2, 174.3, 174.4, 174.5, 174.6, 174.8, 174.9, 175, 175.9, 176, 176.1, 176.2, 176.3, 176.4, 176.5, 176.8, 176.9 |  |
| Malignant Neoplasm Of Genitourinary Organs | 179, 180, 180.1, 180.8, 180.9, 181, 182, 182.1, 182.8, 183, 183.2, 183.3, 183.4, 183.5, 183.8, 183.9, 184, 184.1, 184.2, 184.3, 184.4, 184.8, 184.9, 185, 186, 186.9, 187.1, 187.2, 187.3, 187.4, 187.5, 187.6, 187.7, 187.8, 187.9, 188, 188.1, 188.2, 188.3, 188.4, 188.5, 188.6, 188.7, 188.8, 188.9, 189, 189.1, 189.2, 189.3, 189.4, 189.8, 189.9 |  |
| Malignant Neoplasm Of Other And Unspecified Sites | 190, 190.1, 190.2, 190.3, 190.4, 190.5, 190.6, 190.7, 190.8, 190.9, 191, 191.1, 191.2, 191.3, 191.4, 191.5, 191.6, 191.7, 191.8, 191.9, 192, 192.1, 192.2, 192.3, 192.8, 192.9, 193, 194, 194.1, 194.3, 194.4, 194.5, 194.6, 194.8, 194.9, 195, 195.1, 195.2, 195.3, 195.4, 195.5, 195.8, 196, 196.1, 196.2, 196.3, 196.5, 196.6, 196.8, 196.9, 197, 197.1, 197.2, 197.3, 197.4, 197.5, 197.6, 197.7, 197.8, 198, 198.1, 198.2, 198.3, 198.4, 198.5, 198.6, 198.7, 198.81, 198.82, 198.89, 199, 199.1, 199.2 | C76.0, C76.1, C76.2, C76.3, C76.40, C76.41, C76.42, C76.50, C76.51, C76.52, C76.8, C77.0, C77.1, C77.2, C77.3, C77.4, C77.5, C77.8, C77.9, C78.00, C78.01, C78.02, C78.1, C78.2, C78.30, C78.39, C78.4, C78.5, C78.6, C78.7, C78.80, C78.89, C79.00, C79.01, C79.02, C79.10, C79.11, C79.19, C79.2, C79.31, C79.32, C79.40, C79.49, C79.51, C79.52, C79.60, C79.61, C79.62, C79.70, C79.71, C79.72, C79.81, C79.82, C79.89, C79.9, C80.0, C80.1, C80.2 |
| Malignant Neoplasm Of Lymphatic And Hematopoietic Tissue | 200, 200.01, 200.02, 200.03, 200.04, 200.05, 200.06, 200.07, 200.08, 200.1, 200.11, 200.12, 200.13, 200.14, 200.15, 200.16, 200.17, 200.18, 200.2, 200.21, 200.22, 200.23, 200.24, 200.25, 200.26, 200.27, 200.28, 200.3, 200.31, 200.32, 200.33, 200.34, 200.35, 200.36, 200.37, 200.38, 200.4, 200.41, 200.42, 200.43, 200.44, 200.45, 200.46, 200.47, 200.48, 200.5, 200.51, 200.52, 200.53, 200.54, 200.55, 200.56, 200.57, 200.58, 200.6, 200.61, 200.62, 200.63, 200.64, 200.65, 200.66, 200.67, 200.68, 200.7, 200.71, 200.72, 200.73, 200.74, 200.75, 200.76, 200.77, 200.78, 200.8, 200.81, 200.82, 200.83, 200.84, 200.85, 200.86, 200.87, 200.88, 201, 201.01, 201.02, 201.03, 201.04, 201.05, 201.06, 201.07, 201.08, 201.1, 201.11, 201.12, 201.13, 201.14, 201.15, 201.16, 201.17, 201.18, 201.2, 201.21, 201.22, 201.23, 201.24, 201.25, 201.26, 201.27, 201.28, 201.4, 201.41, 201.42, 201.43, 201.44, 201.45, 201.46, 201.47, 201.48, 201.5, 201.51, 201.52, 201.53, 201.54, 201.55, 201.56, 201.57, 201.58, 201.6, 201.61, 201.62, 201.63, 201.64, 201.65, 201.66, 201.67, 201.68, 201.7, 201.71, 201.72, 201.73, 201.74, 201.75, 201.76, 201.77, 201.78, 201.9, 201.91, 201.92, 201.93, 201.94, 201.95, 201.96, 201.97, 201.98, 202, 202.01, 202.02, 202.03, 202.04, 202.05, 202.06, 202.07, 202.08, 202.1, 202.11, 202.12, 202.13, 202.14, 202.15, 202.16, 202.17, 202.18, 202.2, 202.21, 202.22, 202.23, 202.24, 202.25, 202.26, 202.27, 202.28, 202.3, 202.31, 202.32, 202.33, 202.34, 202.35, 202.36, 202.37, 202.38, 202.4, 202.41, 202.42, 202.43, 202.44, 202.45, 202.46, 202.47, 202.48, 202.5, 202.51, 202.52, 202.53, 202.54, 202.55, 202.56, 202.57, 202.58, 202.6, 202.61, 202.62, 202.63, 202.64, 202.65, 202.66, 202.67, 202.68, 202.7, 202.71, 202.72, 202.73, 202.74, 202.75, 202.76, 202.77, 202.78, 202.8, 202.81, 202.82, 202.83, 202.84, 202.85, 202.86, 202.87, 202.88, 202.9, 202.91, 202.92, 202.93, 202.94, 202.95, 202.96, 202.97, 202.98, 203, 203.01, 203.02, 203.1, 203.11, 203.12, 203.8, 203.81, 203.82, 204, 204.01, 204.02, 204.1, 204.11, 204.12, 204.2, 204.21, 204.22, 204.8, 204.81, 204.82, 204.9, 204.91, 204.92, 205, 205.01, 205.02, 205.1, 205.11, 205.12, 205.2, 205.21, 205.22, 205.3, 205.31, 205.32, 205.8, 205.81, 205.82, 205.9, 205.91, 205.92, 206, 206.01, 206.02, 206.1, 206.11, 206.12, 206.2, 206.21, 206.22, 206.8, 206.81, 206.82, 206.9, 206.91, 206.92, 207, 207.01, 207.02, 207.1, 207.11, 207.12, 207.2, 207.21, 207.22, 207.8, 207.81, 207.82, 208, 208.01, 208.02, 208.1, 208.11, 208.12, 208.2, 208.21, 208.22, 208.8, 208.81, 208.82, 208.9, 208.91, 208.92, 209, 209.01, 209.02, 209.03, 209.1, 209.11, 209.12, 209.13, 209.14, 209.15, 209.16, 209.17, 209.2, 209.21, 209.22, 209.23, 209.24, 209.25, 209.26, 209.27, 209.29, 209.3, 209.31, 209.32, 209.33, 209.34, 209.35, 209.36, 209.7, 209.71, 209.72, 209.73, 209.74, 209.75, 209.79 | C81.00, C81.01, C81.02, C81.03, C81.04, C81.05, C81.06, C81.07, C81.08, C81.09, C81.10, C81.11, C81.12, C81.13, C81.14, C81.15, C81.16, C81.17, C81.18, C81.19, C81.20, C81.21, C81.22, C81.23, C81.24, C81.25, C81.26, C81.27, C81.28, C81.29, C81.30, C81.31, C81.32, C81.33, C81.34, C81.35, C81.36, C81.37, C81.38, C81.39, C81.40, C81.41, C81.42, C81.43, C81.44, C81.45, C81.46, C81.47, C81.48, C81.49, C81.70, C81.71, C81.72, C81.73, C81.74, C81.75, C81.76, C81.77, C81.78, C81.79, C81.90, C81.91, C81.92, C81.93, C81.94, C81.95, C81.96, C81.97, C81.98, C81.99, C82.00, C82.01, C82.02, C82.03, C82.04, C82.05, C82.06, C82.07, C82.08, C82.09, C82.10, C82.11, C82.12, C82.13, C82.14, C82.15, C82.16, C82.17, C82.18, C82.19, C82.20, C82.21, C82.22, C82.23, C82.24, C82.25, C82.26, C82.27, C82.28, C82.29, C82.30, C82.31, C82.32, C82.33, C82.34, C82.35, C82.36, C82.37, C82.38, C82.39, C82.40, C82.41, C82.42, C82.43, C82.44, C82.45, C82.46, C82.47, C82.48, C82.49, C82.50, C82.51, C82.52, C82.53, C82.54, C82.55, C82.56, C82.57, C82.58, C82.59, C82.60, C82.61, C82.62, C82.63, C82.64, C82.65, C82.66, C82.67, C82.68, C82.69, C82.80, C82.81, C82.82, C82.83, C82.84, C82.85, C82.86, C82.87, C82.88, C82.89, C82.90, C82.91, C82.92, C82.93, C82.94, C82.95, C82.96, C82.97, C82.98, C82.99, C83.00, C83.01, C83.02, C83.03, C83.04, C83.05, C83.06, C83.07, C83.08, C83.09, C83.10, C83.11, C83.12, C83.13, C83.14, C83.15, C83.16, C83.17, C83.18, C83.19, C83.30, C83.31, C83.32, C83.33, C83.34, C83.35, C83.36, C83.37, C83.38, C83.39, C83.50, C83.51, C83.52, C83.53, C83.54, C83.55, C83.56, C83.57, C83.58, C83.59, C83.70, C83.71, C83.72, C83.73, C83.74, C83.75, C83.76, C83.77, C83.78, C83.79, C83.80, C83.81, C83.82, C83.83, C83.84, C83.85, C83.86, C83.87, C83.88, C83.89, C83.90, C83.91, C83.92, C83.93, C83.94, C83.95, C83.96, C83.97, C83.98, C83.99, C84.00, C84.01, C84.02, C84.03, C84.04, C84.05, C84.06, C84.07, C84.08, C84.09, C84.10, C84.11, C84.12, C84.13, C84.14, C84.15, C84.16, C84.17, C84.18, C84.19, C84.40, C84.41, C84.42, C84.43, C84.44, C84.45, C84.46, C84.47, C84.48, C84.49, C84.60, C84.61, C84.62, C84.63, C84.64, C84.65, C84.66, C84.67, C84.68, C84.69, C84.70, C84.71, C84.72, C84.73, C84.74, C84.75, C84.76, C84.77, C84.78, C84.79, C84.90, C84.91, C84.92, C84.93, C84.94, C84.95, C84.96, C84.97, C84.98, C84.99, C84.A0, C84.A1, C84.A2, C84.A3, C84.A4, C84.A5, C84.A6, C84.A7, C84.A8, C84.A9, C84.Z0, C84.Z1, C84.Z2, C84.Z3, C84.Z4, C84.Z5, C84.Z6, C84.Z7, C84.Z8, C84.Z9, C85.10, C85.11, C85.12, C85.13, C85.14, C85.15, C85.16, C85.17, C85.18, C85.19, C85.20, C85.21, C85.22, C85.23, C85.24, C85.25, C85.26, C85.27, C85.28, C85.29, C85.80, C85.81, C85.82, C85.83, C85.84, C85.85, C85.86, C85.87, C85.88, C85.89, C85.90, C85.91, C85.92, C85.93, C85.94, C85.95, C85.96, C85.97, C85.98, C85.99, C86.0, C86.1, C86.2, C86.3, C86.4, C86.5, C86.6, C88.0, C88.2, C88.3, C88.4, C88.8, C88.9, C90.00, C90.01, C90.02, C90.10, C90.11, C90.12, C90.20, C90.21, C90.22, C90.30, C90.31, C90.32, C91.00, C91.01, C91.02, C91.10, C91.11, C91.12, C91.30, C91.31, C91.32, C91.40, C91.41, C91.42, C91.50, C91.51, C91.52, C91.60, C91.61, C91.62, C91.90, C91.91, C91.92, C91.A0, C91.A1, C91.A2, C91.Z0, C91.Z1, C91.Z2, C92.00, C92.01, C92.02, C92.10, C92.11, C92.12, C92.20, C92.21, C92.22, C92.30, C92.31, C92.32, C92.40, C92.41, C92.42, C92.50, C92.51, C92.52, C92.60, C92.61, C92.62, C92.90, C92.91, C92.92, C92.A0, C92.A1, C92.A2, C92.Z0, C92.Z1, C92.Z2, C93.00, C93.01, C93.02, C93.10, C93.11, C93.12, C93.30, C93.31, C93.32, C93.90, C93.91, C93.92, C93.Z0, C93.Z1, C93.Z2, C94.00, C94.01, C94.02, C94.20, C94.21, C94.22, C94.30, C94.31, C94.32, C94.40, C94.41, C94.42, C94.6, C94.80, C94.81, C94.82, C95.00, C95.01, C95.02, C95.10, C95.11, C95.12, C95.90, C95.91, C95.92, C96.0, C96.2, C96.4, C96.5, C96.6, C96.9, C96.A, C96.Z |
| Neoplasms Of Uncertain Behavior | 235, 235.1, 235.2, 235.3, 235.4, 235.5, 235.6, 235.7, 235.8, 235.9, 236, 236.1, 236.2, 236.3, 236.4, 236.5, 236.6, 236.7, 236.9, 236.91, 236.99, 237, 237.1, 237.2, 237.3, 237.4, 237.5, 237.6, 237.7, 237.71, 237.72, 237.73, 237.79, 237.9, 238, 238.1, 238.2, 238.3, 238.4, 238.5, 238.6, 238.71, 238.72, 238.73, 238.74, 238.75, 238.76, 238.77, 238.79, 238.8, 238.9 | D37.01, D37.02, D37.030, D37.031, D37.032, D37.039, D37.04, D37.05, D37.09, D37.1, D37.2, D37.3, D37.4, D37.5, D37.6, D37.8, D37.9, D38.0, D38.1, D38.2, D38.3, D38.4, D38.5, D38.6, D39.0, D39.10, D39.11, D39.12, D39.2, D39.8, D39.9, D40.0, D40.10, D40.11, D40.12, D40.8, D40.9, D41.00, D41.01, D41.02, D41.10, D41.11, D41.12, D41.20, D41.21, D41.22, D41.3, D41.4, D41.8, D41.9, D42.0, D42.1, D42.9, D43.0, D43.1, D43.2, D43.3, D43.4, D43.8, D43.9, D44.0, D44.10, D44.11, D44.12, D44.2, D44.3, D44.4, D44.5, D44.6, D44.7, D44.9, D45, D46.0, D46.1, D46.20, D46.21, D46.22, D46.4, D46.9, D46.A, D46.B, D46.C, D46.Z, D47.0, D47.1, D47.2, D47.3, D47.4, D47.9, D47.Z1, D47.Z2, D47.Z9, D48.0, D48.1, D48.2, D48.3, D48.4, D48.5, D48.60, D48.61, D48.62, D48.7, D48.9, Q85.00, Q85.01, Q85.02, Q85.03, Q85.09 |
| Neoplasms of Unspecified Nature | 239, 239.1, 239.2, 239.3, 239.4, 239.5, 239.6, 239.7, 239.81, 239.89, 239.9 | D49.0, D49.1, D49.2, D49.3, D49.4, D49.5, D49.511, D49.512, D49.519, D49.59, D49.6, D49.7, D49.81, D49.89, D49.9 |
| Malignant neoplasms of bone and articular cartilage |  | C40.00, C40.01, C40.02, C40.10, C40.11, C40.12, C40.20, C40.21, C40.22, C40.30, C40.31, C40.32, C40.80, C40.81, C40.82, C40.90, C40.91, C40.92, C41.0, C41.1, C41.2, C41.3, C41.4, C41.9 |
| Melanoma and other malignant neoplasms of skin |  | C43.0, C43.10, C43.11, C43.12, C43.20, C43.21, C43.22, C43.30, C43.31, C43.39, C43.4, C43.51, C43.52, C43.59, C43.60, C43.61, C43.62, C43.70, C43.71, C43.72, C43.8, C43.9, C4A.0, C4A.10, C4A.11, C4A.12, C4A.20, C4A.21, C4A.22, C4A.30, C4A.31, C4A.39, C4A.4, C4A.51, C4A.52, C4A.59, C4A.60, C4A.61, C4A.62, C4A.70, C4A.71, C4A.72, C4A.8, C4A.9 |
| Malignant neoplasms of mesothelial and soft tissue |  | C45.0, C45.1, C45.2, C45.7, C45.9, C46.0, C46.1, C46.2, C46.3, C46.4, C46.50, C46.51, C46.52, C46.7, C46.9, C47.0, C47.10, C47.11, C47.12, C47.20, C47.21, C47.22, C47.3, C47.4, C47.5, C47.6, C47.8, C47.9, C48.0, C48.1, C48.2, C48.8, C49.0, C49.10, C49.11, C49.12, C49.20, C49.21, C49.22, C49.3, C49.4, C49.5, C49.6, C49.8, C49.9, C49.A0, C49.A1, C49.A2, C49.A3, C49.A4, C49.A5, C49.A9 |
| Malignant neoplasms of breast |  | C50.011, C50.012, C50.019, C50.021, C50.022, C50.029, C50.111, C50.112, C50.119, C50.121, C50.122, C50.129, C50.211, C50.212, C50.219, C50.221, C50.222, C50.229, C50.311, C50.312, C50.319, C50.321, C50.322, C50.329, C50.411, C50.412, C50.419, C50.421, C50.422, C50.429, C50.511, C50.512, C50.519, C50.521, C50.522, C50.529, C50.611, C50.612, C50.619, C50.621, C50.622, C50.629, C50.811, C50.812, C50.819, C50.821, C50.822, C50.829, C50.911, C50.912, C50.919, C50.921, C50.922, C50.929 |
| Malignant neoplasms of female genital organs |  | C51.0, C51.1, C51.2, C51.8, C51.9, C52, C53.0, C53.1, C53.8, C53.9, C54.0, C54.1, C54.2, C54.3, C54.8, C54.9, C55, C56.1, C56.2, C56.9, C57.00, C57.01, C57.02, C57.10, C57.11, C57.12, C57.20, C57.21, C57.22, C57.3, C57.4, C57.7, C57.8, C57.9, C58 |
| Malignant neoplasms of male genital organs |  | C60.0, C60.1, C60.2, C60.8, C60.9, C61, C62.00, C62.01, C62.02, C62.10, C62.11, C62.12, C62.90, C62.91, C62.92, C63.00, C63.01, C63.02, C63.10, C63.11, C63.12, C63.2, C63.7, C63.8, C63.9 |
| Malignant neoplasms of urinary tract |  | C64.1, C64.2, C64.9, C65.1, C65.2, C65.9, C66.1, C66.2, C66.9, C67.0, C67.1, C67.2, C67.3, C67.4, C67.5, C67.6, C67.7, C67.8, C67.9, C68.0, C68.1, C68.8, C68.9 |
| Malignant neoplasms of eye, brain and other parts of central nervous system |  | C69.00, C69.01, C69.02, C69.10, C69.11, C69.12, C69.20, C69.21, C69.22, C69.30, C69.31, C69.32, C69.40, C69.41, C69.42, C69.50, C69.51, C69.52, C69.60, C69.61, C69.62, C69.80, C69.81, C69.82, C69.90, C69.91, C69.92, C70.0, C70.1, C70.9, C71.0, C71.1, C71.2, C71.3, C71.4, C71.5, C71.6, C71.7, C71.8, C71.9, C72.0, C72.1, C72.20, C72.21, C72.22, C72.30, C72.31, C72.32, C72.40, C72.41, C72.42, C72.50, C72.59, C72.9 |
| Malignant neoplasms of thyroid and other endocrine glands |  | C73, C74.00, C74.01, C74.02, C74.10, C74.11, C74.12, C74.90, C74.91, C74.92, C75.0, C75.1, C75.2, C75.3, C75.4, C75.5, C75.8, C75.9 |
| Malignant neuroendocrine tumors |  | C7A.00, C7A.010, C7A.011, C7A.012, C7A.019, C7A.020, C7A.021, C7A.022, C7A.023, C7A.024, C7A.025, C7A.026, C7A.029, C7A.090, C7A.091, C7A.092, C7A.093, C7A.094, C7A.095, C7A.096, C7A.098, C7A.1, C7A.8 |
| Secondary neuroendocrine tumors |  | C7B.00, C7B.01, C7B.02, C7B.03, C7B.04, C7B.09, C7B.1, C7B.8 |

**Appendix Table 3. Opioid use disorder (OUD) and opioid overdose: Definition by International Classification of Disease (ICD9 and ICD10 codes)**

| **Outcome** | **ICD-9-CM codes** | **ICD-10-codes** |
| --- | --- | --- |
| Opioid use disorder | 304.0, 304.00, 304.01, 304.02, 304.7, 304.70, 304.71, 304.72, 305.5, 305.50, 305.51, 305.52, 760.72, 965.0, 965.00, 965.01, 965.02, 965.09, E85.00, E85.01, E85.02, E93.50, E93.51 | DX F11.10, F11.120, F11.121, F11.122, F11.129, F11.13, F11.14, F11.150, F11.151, F11.159, F11.181, F11.182, F11.188, F11.19, F11.20, F11.220, F11.221, F11.222, F11.229, F11.23, F11.24, F11.250, F11.251, F11.259, F11.281, F11.282, F11.288, F11.29, F11.90, F11.920, F11.921, F11.922, F11.929, F11.93, F11.94, F11.950, F11.951, F11.959, F11.981, F11.982, F11.988, F11.99, T40.0X1A, T40.0X2A, T40.0X3A, T40.0X4A, T40.1X1A, T40.1X2A, T40.1X3A, T40.1X4A, T40.2X1A, T40.2X2A, T40.2X3A, T40.2X4A, T40.3X1A, T40.3X2A, T40.3X3A, T40.3X4A, T40.3X5A, T40.4X1A, T40.4X2A, T40.4X3A, T40.4X4A, T40.411A, T40.412A, T40.413A, T40.414A, T40.415A, T40.421A, T40.422A, T40.423A, T40.424A, T40.425A, T40.491A, T40.492A, T40.493A, T40.494A, T40.495A, T40.601A, T40.602A, T40.603A, T40.604A, T40.691A, T40.692A, T40.693A, T40.694A |
| Opioid-related poisoning | 965.00, 965.01, 965.02, 965.09, E850.0, E850.1, E850.2, E950.0, E980.0, X42, X62, Y12 | T40.0X1, T40.0X3, T40.0X4, T40.0X5, T40.1X1, T40.1X3, T40.1X4, T40.2X1, T40.2X3, T40.2X4, T40.2X5, T40.3X1, T40.3X3, T40.3X4, T40.3X5, T40.4X1, T40.4X3, T40.4X4, T40.4X5, T40.601, T40.603, T40.604, T40.605, T40.691, T40.693, T40.694, T40.695 |
| Opioid-related adverse events | E935.0, E935.1, E935.2 | Y45.0 |
| Opioid overdose | 276.4, 292.1, 292.81, 292.8, 486, 496, 518.81, 518.82, 780.0, 780.97, 786.03, 786.05, 786.09, 786.52, 799.0, E950, E951, E952, E953, E954, E955, E956, E957, E958, E959 | E87.4, F19.950, F19.951, F19.921, F19.97, F19.96, F19.94, F11.182, F11.181, J18.9, J44.9, J96.90, J80, R40.20, R40.3, R40.4, R40.0, R41.82, R06.81, R06.02, R06.00, R07.1, R09.01, R09.02, T40.0, T40.1, T40.2, T40.3, T40.4 |

**Appendix Table 4: Diagnosis codes used to identify substance use history**

| **History of substance abuse and/or substance use disorder** | **Code type** | **ICD-9 and ICD-10 codes** |
| --- | --- | --- |
| Cannabis-related disorders | ICD-10 | F12.10, F12.11, F12.120, F12.121, F12.122, F12.129, F12.13, F12.150, F12.151, F12.159, F12.180, F12.188, F12.19, F12.20, F12.21, F12.220, F12.221, F12.222, F12.229, F12.23, F12.250, F12.251, F12.259, F12.280, F12.288, F12.29, F12.90, F12.91, F12.920, F12.921, F12.922, F12.929, F12.93, F12.950, F12.951, F12.959, F12.980, F12.988, F12.99 |
|  | ICD-9 | 304.30, 304.31, 304.32, 304.33, 305.20, 305.21, 305.22, 305.23 |
| Sedative, hypnotic, or anxiolytic related disorders | ICD-10 | F13.10, F13.11, F13.120, F13.121, F13.129, F13.130, F13.131, F13.132, F13.139, F13.14, F13.150, F13.151, F13.159, F13.180, F13.181, F13.182, F13.188, F13.19, F13.20, F13.21, F13.20, F13.221, F13.229, F13.230, F13.231, F13.232, F13.239, F13.24, F13.250, F13.251, F13.259, F13.26, F13.27,, F13.280, F13.281, F13.282, F13.288, F13.29, F13.90, F13.91, F13.920, F13.921, F13.929, F13.930, F13.931, F13.932, F13.939, F13.94, F13.950, F13.951, F13.959, F13.96, F13.97, F13.980, F13.981, F13.982, F13.988, F13.99, |
|  | ICD-9 | 304.10, 304.11, 304.12, 304.13, 305.40, 305.41, 305.42, 305.43 |
| Cocaine related disorders | ICD-10 | F14.10, F14.11, F14.120, F14.121, F14.122, F14.129, F14.13, F14.14, F14.150, F14.151, F14.159, F14.180, F14.181, F14.182, F14.188, F14.19, F14.20, F14.21, F14.220, F14.221, F14.222, F14.229, F14.23, F14.24, F14.250, F14.251, F14.259, F14.280, F14.281, F14.282, F14.288, F14.29, F14.90, F14.91, F14.920, F14.921, F14.922, F14.929, F14.93, F14.94, F14.950, F14.951, F14.959, F14.980, F14.981, F14.982, F14.988, F14.99, |
|  | ICD-9 | 304.20, 304.21, 304.22, 304.23, 305.60, 305.61, 305.62, 305.63 |
| Other stimulant related disorders | ICD-10 | F15.10, F15.11, F15.120, F15.121, F15.122, F15.129, F15.13, F15.14, F15.150, F15.151, F15.159, F15.180, F15.181, F15.182, F15.188, F15.19, F15.20, F15.21, F15.220, F15.221, F15.222, F15.229, F15.23, F15.24, F15.250, F15.251, F15.259, F15.280, F15.281, F15.282, F15.288, F15.29, F15.90, F15.91, F15.920, F15.921, F15.922, F15.929, F15.93, F15.94, F15.950, F15.951, F15.959, F15.980, F15.981, F15.982, F15.988, F15.99 |
|  | ICD-9 | 304.40, 304.41, 304.42, 304.43, 305.70, 305.71, 305.72, 305.73 |
| Hallucinogen related disorders | ICD-10 | F16.10, F16.11, F16.120, F16.121, F16.122, F16.129, F16.14, F16.150, F16.151, F16.159, F16.180, F16.183, F16.188, F16.19, F16.20, F16.21, F16.220, F16.221, F16.229, F16.24, F16.250, F16.251, F16.259, F16.280, F16.283, F16.288, F16.29, F16.90, F16.91, F16.920, F16.921, F16.929, F16.94, F16.950, F16.951, F16.959, F16.980, F16.983, F16.988, F16.99 |
|  | ICD-9 | 304.50, 304.51, 304.52, 304.53, 305.30, 305.31, 305.32, 305.33 |
| Inhalant related disorders |  | F18.10, F18.11, F18.120, F18.121, F18.122, F18.129, F18.14, F18.150, F18.151, F18.159, F18.180, F18.183, F18.188, F18.19, F18.20, F18.21, F18.220, F18.221, F18.229, F18.24, F18.250, F18.251, F18.259, F18.27, F18.280, F18.283, F18.288, F18.29, F18.90, F18.91, F18.920, F18.921, F18.929, F18.94, F18.950, F18.951, F18.959, F18.97, F18.980, F18.983, F18.988, F18.99 |
| Other psychoactive substance related disorders | ICD-10 | F19.10, F19.11, F19.120, F19.121, F19.122, F19.129, F19.14, F19.150, F19.151, F19.159, F19.180, F19.183, F19.188, F19.19, F19.20, F19.21, F19.220, F19.221, F19.229, F19.24, F19.250, F19.251, F19.259, F19.27, F19.280, F19.283, F19.288, F19.29, F19.90, F19.91, F19.920, F19.921, F19.929, F19.94, F19.950, F19.951, F19.959, F19.97, F19.980, F19.983, F19.988, F19.99 |
|  | ICD-9 | 304.60, 304.61, 304.62, 304.63, 304.80, 304.81, 304.82, 304.83, 304.90, 304.91, 304.92, 304.93, 305.80, 305.81, 305.82, 305.83, 305.90, 305.91, 305.92, 305.93 |
| Nicotine dependence | ICD-10 | F17.200, F17.201, F17.203, F17.208, F17.209, F17.210, F17.211, F17.213, F17.218, F17.219, F17.220, F17.221, F17.223, F17.228, F17.229, F17.290, F17.291, F17.293, F17.298, F17.299 |
| Alcohol related disorders | ICD-10 | F10.10, F10.11, F10.120, F10.121, F10.129, F10.130, F10.131, F10.132, F10.139, F10.14, F10.150, F10.151, F10.159, F10.180, F10.181, F10.182, F10.188, F10.19, F10.20, F10.21, F10.220, F10.221, F10.229, F10.230, F10.231, F10.232, F10.239, F10.24, F10.250, F10.251, F10.259, F10.26, F10.27, F10.280, F10.281, F10.282, F10.288, F10.29, F10.90, F10.91, F10.920, F10.921, F10.929, F10.930, F10.931, F10.932, F10.939, F10.94, F10.950, F10.951, F10.959, F10.96, F10.97, F10.980, F10.981, F10.982, F10.988, F10.99 |
| Abuse of non-psychoactive substances | ICD-10 | F55.0, F55.1, F55.2, F55.3, F55.4, F55.8, |
| Alcohol-induced mental disorders | ICD-9 | 291.0, 291.1, 291.2, 291.3, 291.4, 291.5, 291.81, 291.82, 291.89, 291.9, |
| Alcohol dependence syndrome | ICD-9 | 303.00, 303.01, 303.02, 303.03, 303.90, 303.91, 303.92, 303.93, |
| Drug-induced mental disorders | ICD-9 | 292.0, 292.11, 292.12, 292.2, 292.81, 292.82, 292.83, 292.84, 292.85, 292.89, 292.9, |
| Tobacco use | ICD-10 | Z72.0, |
| Tobacco use disorder | ICD-9 | 305.1 |
| Opioid type dependence | ICD-9 | 304.0,0, 304.01, 304.02, 304.03 |
| Combinations of opioid type drug with any other drug dependence | ICD-9 | 304.70, 304.71, 304.72, 304.73 |
| Non-dependent opioid abuse | ICD-9 | 305.50, 305.51, 305.52, 305.53 |
| Non-dependent alcohol abuse | ICD-9 | 305.00, 305.01, 305.02, 305.03 |
| Alcoholic polyneuropathy | ICD-9 | 357.5 |
|  | ICD-10 | G62.1 |
| Alcoholic cardiomyopathy | ICD-9 | 425.5 |
|  | ICD-10 | I42.6 |
| Alcoholic gastritis | ICD-9 | 535.30, 535.31 |
|  | ICD-10 | K29.20, K29.21 |
| Alcoholic hepatitis | ICD-10 | K70.10, K70.11 |
| Chronic liver disease and cirrhosis | ICD-9 | 571.0, 571.1, 571.2, 571.3 |
|  | ICD-10 | K70.30, K70.31 |
| Toxic effect of ethyl alcohol | ICD-9 | 980.0 |
| Drug dependence complicating pregnancy childbirth or the puerperium | ICD-9 | 648.30, 648.31, 648.32, 648.33, 648.34 |
| Suspected damage to fetus from drugs affecting management of mother | ICD-9 | 655.50, 655.51, 655.53 |
| Drug withdrawal syndrome in newborn | ICD-9 | 779.5 |
|  | ICD-10 | P96.1, P96.2 |
| Alcoholic fibrosis and sclerosis of liver | ICD-10 | K70.2 |
| Alcoholic hepatic failure | ICD-10 | K70.40, K70.41 |
| Alcoholic liver disease | ICD-10 | K70.9 |
| Newborn affected by maternal use of alcohol | ICD-10 | P04.3 |
| Fetal alcohol syndrome | ICD-10 | Q86.0 |
| Unspecified opioid use | ICD-10 | F11.90, F11.91, F11.920, F11.921, F11.922, F11.929, F11.93, F11.94, F11.950, F11.951, F11.959, F11.981, F11.982, F11.988, F11.99 |
| Poisoning by, adverse effect of and underdosing of cannabis | ICD-10 | T40.711A, T40.711D, T40.711S, T40.712A, T40.712D, T40.712S, T40.713A, T40.713D, T40.713S, T40.714A, T40.714D, T40.714S, T40.715A, T40.715D, T40.715S, T40.716A, T40.716D, T40.716S, T40.721A, T40.721D, T40.721S, T40.722A, T40.722D, T40.722S, T40.723A, T40.723D, T40.723S, T40.724A, T40.724D, T40.724S, T40.725A, T40.725D, T40.725S, T40.726A, T40.726D, T40.726S |
| Poisoning by and adverse effect of lysergide (LSD) | ICD-10 | T40.8X1A, T40.8X1D, T40.8X1S, T40.8X2Z, T40.8X2D, T40.8X2S, T40.8X3A, T40.8X3D, T40.8X3S, T40.8X4A, T40.8X4D, T40.8X4S |
| Alcohol use complicating pregnancy, childbirth, and the puerperium | ICD-10 | O99.310, O99.311, O99.312, O99.313, O99.314, O99.315 |
| Drug use complicating pregnancy, childbirth, and the puerperium | ICD-10 | O99.320, O99.321, O99.322, O99.323, O99.324, O99.325 |
| Newborn affected by maternal use of cocaine | ICD-10 | P04.41 |
| Newborn affected by maternal use of other drugs of addiction | ICD-10 | P04.49 |
| Poisoning by, adverse effect of and underdosing of cocaine | ICD-10 | T40.711A, T40.711D, T40.711S, T40.712A, T40.712D, T40.712S, T40.713A, T40.713D, T40.713S, T40.714A, T40.714D, T40.714S, T40.715A, T40.715D, T40.715S, T40.716A, T40.716D, T40.716S, |
| Personal history of tobacco use | ICD-9 | V15.82 |
| Noxious influences affecting fetus or newborn via placenta or breast milk | ICD-9 | 760.2, 760.3, 760.5 |
| Counseling on substance use and abuse | ICD-9 | V65.42 |
| Maternal care for (suspected) damage to fetus by drugs | ICD-10 | O35.5XX0, O35.5XX1, O35.5XX2, O35.5XX3, O35.5XX4, O35.5XX5, O35.5XX9 |
| Adverse effect and underdosing of opium | ICD-10 | T40.0X5A, T40.0X5D, T40.0X5S,  T40.0X6A, T40.0X6D, T40.0X6S |
| Poisoning by, adverse effect of and underdosing of unspecified psychodysleptics [hallucinogens] | ICD-10 | T40.901A, T40.901D, T40.901S,  T40.902A, T40.902D, T40.902S, T40.903A, T40.903D, T40.903S, T40.904A, T40.904D, T40.904S, T40.905A, T40.905D, T40.905S, T40.906A, T40.906D, T40.906S |
| Poisoning by, adverse effect of and underdosing of other psychodysleptics [hallucinogens] | ICD-10 | T40.991A, T40.991D, T40.991S,  T40.992A, T40.992D, T40.992S, T40.993A, T40.993D, T40.993S, T40.994A, T40.994D, T40.994S, T40.995A, T40.995D, T40.995S, T40.996A, T40.996D, T40.996S |
| Personal history of nicotine dependence | ICD-10 | Z87.891 |

**Appendix 5: Sensitivity analysis 1**

**Methods**: For sensitivity analysis 1, beneficiaries with substance use in the 6-month baseline period, identified using a combination of ICD-9 and ICD-10 codes (Appendix Table 4), were excluded from the final study cohort (substance-naïve) in the model predicting incidence of OUD.

**Results:** After this exclusion, the relationship between opioid tapering and risk of OUD was assessed. From the final cohort, 3,617 OUD cases and 7,234 matched controls were identified. After adjusting for covariates, compared with steady dose, the odds of OUD were significantly lower for slow tapering (aOR=0.84; 95%CI: 0.72-0.98) and rapid tapering (aOR=0.71; 95%CI: 0.60-0.85). These results are consistent with the primary analysis for findings regarding rapid tapering.

**Appendix Table 5**: Adjusted relationship between rate of opioid dose changes and OUD among substance-use naïve patients on long-term opioid therapy

| Patient Characteristics | Adjusted odds  ratio (95% CI) | p-value |
| --- | --- | --- |
| Rate of opioid dose changes |  |  |
| Steady dose | Reference |  |
| Slow tapering | 0.835 (0.715 - 0.975) | 0.022 |
| Rapid tapering | 0.714 (0.599 - 0.851) | <0.001 |
| Dose escalation | 1.062 (0.924 - 1.220) | 0.397 |
| Gender |  |  |
| Female | 1.074 (0.970 - 1.189) | 0.172 |
| Race and ethnicity |  |  |
| Non-Hispanic White | Reference |  |
| Black | 0.906 (0.777 - 1.057) | 0.208 |
| Hispanic | 0.964 (0.792 - 1.172) | 0.711 |
| Other racial groups | 0.982 (0.769 - 1.252) | 0.881 |
| LIS enrollment |  |  |
| Yes | 0.912 (0.830 - 1.002) | 0.055 |
| Multiple CNCP | 1.375 (1.253 - 1.509) | <0.001 |
| Renal impairment | 1.127 (0.970 - 1.310) | 0.118 |
| Hepatic impairment | 0.800 (0.534 - 1.200) | 0.281 |
| Sleep disorders | 1.223 (1.089 - 1.373) | <0.001 |
| Parkinson | 1.360 (0.980 - 1.887) | 0.065 |
| Mental disorder | 0.638 (0.553 - 0.737) | <0.001 |
| COPD | 0.374 (0.320 - 0.437) | <0.001 |
| CCI | 1.439 (1.313 - 1.576) | <0.001 |
| 0 |  |  |
| 1-2 | Reference |  |
| >=3 | 1.822 (1.638 - 2.026) | <0.001 |
| Mean opioid daily dose during baseline hazard  period (MME) | 3.284 (2.701 - 3.992) | <0.001 |
| <20 | Reference |  |
| 20 – 50 | 1.750 (1.559 - 1.966) | <0.001 |
| >= 50 | 3.580 (3.132 - 4.093) | <0.001 |
| Abbreviations: LIS: Low-Income Status; COPD: Chronic Obstructive Pulmonary Disease; CCI: Deyo-Charlson Comorbidity Index; CNCP: Chronic Non-Cancer Pain; MME: Morphine Milligram Equivalents; CI: Confidence Interval | | |

**Appendix 6:** **Sensitivity analysis 2**

**Methods:** To focus on new long-term opioid users, sensitivity analysis 2 was conducted focusing on a cohort of older Medicare beneficiaries with chronic non-cancer pain who initiated a new course of LTOT between January 1^st^, 2014 – October 31^th^, 2020. Beneficiaries were required to have at least two years of history with no opioid fills in order to be included in this analysis.

**Results:** Sensitivity analysis 2 identified a cohort of 42,178 patients. Among them, 478 cases for OD, 1,644 cases of OUD and 4,315 cases of mortality were identified. After controlling for patient socio-demographics and clinical characteristics, compared with steady dose, the odds of OD were significantly lower (aOR=0.40; 95%CI=0.25-0.63) for rapid tapering and significantly higher (aOR=2.9; 95%CI=1.93-4.3) for dose escalation. Compared with steady dose, the odds of OUD were significantly lower (aOR=0.51; 95%CI=0.41-0.64) for rapid tapering and significantly higher (aOR=1.72; 95%CI=1.4-2.12) for dose escalation. Compared to steady dose, significantly higher odds for all-cause mortality were found among patients undergoing tapering (aOR=1.49; 95%CI=1.3-1.71), rapid tapering (aOR=2.03; 95%CI=1.8-2.3), and dose escalation (aOR=1.88; 95%CI=1.63-2.16). These results were consistent with our primary analyses across all three study outcomes, indicating that the impact of opioid use history was likely minimal.

**Appendix Table 6**: Adjusted relationship between rate of opioid dose changes and OD, OUD, and all-cause mortality among new patients on long-term opioid therapy

| Patient Characteristics | Overdose | | Opioid use disorder | | All-cause Mortality | |
| --- | --- | --- | --- | --- | --- | --- |
|  | Adjusted Odds ratio (95% CI) | p-value | Adjusted Odds ratio (95% CI) | p-value | Adjusted Odds ratio (95% CI) | p-value |
| Rate of opioid dose changes |  |  |  |  |  |  |
| Steady dose | Reference |  | Reference |  | Reference |  |
| Slow Tapering | 1.25  (0.818 - 1.91) | 0.301 | 0.836  (0.664 - 1.052) | 0.127 | 1.492  (1.297 - 1.717) | <0.001 |
| Rapid tapering | 0.398  (0.251 - 0.631) | <0.001 | 0.511  (0.408 - 0.639) | <0.001 | 2.030  (1.798 - 2.291) | <0.001 |
| Escalation | 2.879  (1.931 - 4.292) | <0.001 | 1.720  (1.393 - 2.124) | <0.001 | 1.879  (1.633 - 2.162) | <0.001 |
| Gender |  |  |  |  |  |  |
| Female | 0.989  (0.747 - 1.31) | 0.939 | 1.001  (0.867 - 1.155) | 0.994 | 0.796  (0.716 - 0.884) | <0.001 |
| Race and ethnicity |  |  |  |  |  |  |
| Non-Hispanic White | Reference |  | Reference |  | Reference |  |
| Black | 0.755  (0.476 - 1.197) | 0.233 | 1.039  (0.834 - 1.294) | 0.733 | 0.727  (0.617 - 0.858) | <0.001 |
| Hispanic | 0.757  (0.276 - 2.078) | 0.589 | 1.088  (0.703 - 1.684) | 0.705 | 0.38  (0.274 - 0.529) | <0.001 |
| Other racial groups | 0.716  (0.371 - 1.384) | 0.321 | 0.873  (0.596 - 1.278) | 0.485 | 0.495  (0.372 - 0.66) | <0.001 |
| LIS enrollment |  |  |  |  |  |  |
| Yes | 0.688  (0.52 - 0.911) | 0.009 | 0.907  (0.791 - 1.039) | 0.160 | 1.592  (1.451 - 1.746) | <0.001 |
| Multiple CNCP | 1.818  (1.389 - 2.378) | <0.001 | 1.428  (1.252 - 1.629) | <0.001 | 0.843  (0.769 - 0.925) | <0.001 |
| Renal impairment | 1.127  (0.756 - 1.679) | 0.557 | 0.910  (0.734 - 1.128) | 0.390 | 1.640  (1.468 - 1.833) | <0.001 |
| Hepatic impairment | 0.772  (0.19 - 3.138) | 0.718 | 1.651  (0.876 - 3.113) | 0.121 | 1.523  (1.078 - 2.153) | 0.017 |
| Substance use | 1.208(0.897 - 1.626) | 0.214 | 1.204  (1.040 - 1.394) | 0.013 | 1.557  (1.4 - 1.731) | <0.001 |
| Sleep disorders | 1.161  (0.847 - 1.590) | 0.353 | 1.099  (0.935 - 1.292) | 0.251 | 1.866  (1.682 - 2.069) | <0.001 |
| Parkinson | 0.502  (0.223 - 1.129) | 0.096 | 0.879  (0.570 - 1.356) | 0.559 | 1.680  (1.355 - 2.084) | <0.001 |
| Mental disorder | 3.231  (1.336 - 7.816) | 0.009 | 1.522  (1.115 - 2.078) | 0.008 | 1.365  (1.124 - 1.658) | 0.034 |
| COPD | 0.621  (0.199 - 1.931) | 0.410 | 0.793  (0.524 - 1.200) | 0.273 | 1.306  (1.020 - 1.672) |  |
| Concomitant medication use | 1.116  (0.861 - 1.447) | 0.405 | 1.029  (0.904 - 1.171) | 0.670 | 1.147  (1.047 - 1.256) | <0.001 |
| CCI |  |  |  |  |  |  |
| Zero | Reference |  | Reference |  | Reference |  |
| 1_2 | 1.075  (0.752 - 1.537) | 0.692 | 0.899  (0.763 - 1.06) | 0.206 | 3.953  (3.178 - 4.917) | <0.001 |
| >=3 | 2.007  (1.375 - 2.930) | <0.001 | 0.968  (0.814 - 1.151) | 0.713 | 9.083  (7.334 - 11.250) | <0.001 |
| Baseline Opioid mean daily dose (MME) |  |  |  |  |  |  |
| <20 | Reference |  | Reference |  | Reference |  |
| 20 – 50 | 2.153  (1.588 - 2.919) | <.0001 | 1.633  (1.413 - 1.889) | <0.001 | 0.956  (0.868 - 1.052) | 0.357 |
| >= 50 | 3.821  (2.579 - 5.660) | <.0001 | 2.444  (2.010 - 2.971) | <0.001 | 1.252  (1.083 - 1.447) | <0.001 |
| Abbreviations: LIS: Low-Income Status; COPD: Chronic Obstructive Pulmonary Disease; CCI: Deyo-Charlson Comorbidity Index; CNCP: Chronic Non-Cancer Pain; MME: Morphine Milligram. | | | | | | |

**Appendix 7:** **Sensitivity analysis 3**

**Methods:** To focus on individuals who received LTOT for a longer duration of time, , a sensitivity analysis was conducted focusing on a cohort of older Medicare beneficiaries with chronic non-cancer pain who were on LTOT at least one year without opioid discontinuation. Discontinuation was defined as 90 days without opioids possession. The 365^th^ day after first fill of opioid medications was defined as the “index date”.

**Results:** Sensitivity analysis 3 identified a cohort of 77,013 patients. Among them, 1,705 cases for OD, 6,334 cases of OUD and 8,696 cases of mortality were identified. After controlling for patient socio-demographics and clinical characteristics, compared to older adults on a steady dose, the odds of experiencing OD were statistically higher (aOR=2.2; 95%CI=1.73-2.8) for dose escalation**.** Compared with steady dose, the odds of OUD were significantly higher (aOR=1.51; 95%CI=1.35-1.70) for dose escalation. Compared to steady dose, significantly higher odds for all-cause mortality were found among patients undergoing slow tapering (aOR=1.48; 95%CI=1.32-1.65), rapid tapering (aOR=3.3; 95%CI=2.97-3.67), and dose escalation (aOR=1.78; 95%CI=1.60-2.01). While some changes were observed in the significance of the findings, the direction and implications of this analysis are still consistent with our primary analysis. These analyses reveal that heterogeneity in LTOT characteristics is not likely to impact the safety of rapid opioid tapering.

**Appendix Table 7**: Adjusted relationship between rate of opioid dose changes and OD, OUD, and all-cause mortality among patients on long-term opioid therapy for at least a year

| Patient Characteristics | Overdose | | Opioid use disorder | | All-cause Mortality | |
| --- | --- | --- | --- | --- | --- | --- |
|  | Adjusted Odds ratio (95% CI) | p-value | Adjusted Odds ratio (95% CI) | p-value | Adjusted Odds ratio (95% CI) | p-value |
| Rate of opioid dose changes |  |  |  |  |  |  |
| Steady dose | Reference |  | Reference |  | Reference |  |
| Slow Tapering | 0.935  (0.72 - 1.213) | 0.611 | 1.045  (0.927 - 1.179) | 0.471 | 1.476  (1.322 - 1.648) | <0.001 |
| Rapid tapering | 0.950  (0.708 - 1.276) | 0.736 | 0.873  (0.751 - 1.015) | 0.077 | 3.305  (2.975 - 3.671) | <0.001 |
| Escalation | 2.199  (1.729 - 2.798) | <0.001 | 1.517  (1.351 - 1.705) | <0.001 | 1.797  (1.604 - 2.014) | <0.001 |
| Gender |  |  |  |  |  |  |
| Female | 1.149  (0.988 - 1.337) | 0.071 | 1.013  (0.944 - 1.086) | 0.721 | 0.827  (0.767 - 0.893) | <0.001 |
| Race and ethnicity |  |  |  |  |  |  |
| Non-Hispanic White | Reference |  | Reference |  | Reference |  |
| Black | 0.948  (0.746 - 1.205) | 0.664 | 1.017  (0.91 - 1.135) | 0.771 | 0.696  (0.619 - 0.783) | <0.001 |
| Hispanic | 0.841  (0.418 - 1.692) | 0.628 | 0.806  (0.597 - 1.087) | 0.157 | 0.728  (0.562 - 0.943) | 0.016 |
| Other racial groups | 1.567  (1.041 - 2.36) | 0.031 | 0.951  (0.787 - 1.149) | 0.602 | 0.692  (0.563 - 0.85) | <0.001 |
| LIS enrollment |  |  |  |  |  |  |
| Yes | 0.891  (0.775 - 1.026) | 0.108 | 1.018  (0.951 - 1.089) | 0.612 | 1.459  (1.366 - 1.559) | <0.001 |
| Multiple CNCP | 1.882  (1.633 - 2.168) | <0.001 | 1.473  (1.377 - 1.577) | <0.001 | 0.825  (0.772 - 0.882) | <0.001 |
| Renal impairment | 1.077  (0.873 - 1.328) | 0.488 | 1.005  (0.901 - 1.121) | 0.927 | 1.497  (1.38 - 1.623) | <0.001 |
| Hepatic impairment | 0.868  (0.468 - 1.61) | 0.654 | 0.946  (0.711 - 1.259) | 0.703 | 1.309  (1.039 - 1.647) | 0.022 |
| Substance use | 1.638  (1.419 - 1.889) | <0.001 | 1.137  (1.058 - 1.221) | <0.001 | 1.734  (1.608 - 1.871) | <0.001 |
| Sleep disorders | 1.473  (1.256 - 1.726) | <0.001 | 1.072  (0.988 - 1.162) | 0.094 | 2.068  (1.918 - 2.229) | <0.001 |
| Parkinson | 0.803  (0.525 - 1.23) | 0.314 | 0.795  (0.633 - 1) | 0.050 | 1.553  (1.328 - 1.816) | <0.001 |
| Mental disorder | 1.886  (1.387 - 2.564) | <0.001 | 1.309  (1.147 - 1.492) | <0.001 | 1.241  (1.1 - 1.402) | <0.001 |
| COPD | 1.125  (0.755 - 1.674) | 0.563 | 1.057  (0.882 - 1.267) | 0.547 | 1.775  (1.526 - 2.066) | <0.001 |
| Concomitant medication use | 1.306  (1.126 - 1.515) | <0.001 | 1.076  (1.006 - 1.151) | 0.033 | 1.164  (1.088 - 1.245) | <0.001 |
| CCI |  |  |  |  |  |  |
| Zero | Reference |  | Reference |  | Reference |  |
| 1_2 | 1.15  (0.957 - 1.381) | 0.136 | 1.045  (0.964 - 1.134) | 0.285 | 2.602  (2.287 - 2.96) | <0.001 |
| >=3 | 1.479  (1.22 - 1.793) | <0.001 | 1.055  (0.964 - 1.154) | 0.247 | 6.125  (5.395 - 6.954) | <0.001 |
| Baseline Opioid mean daily dose (MME) |  |  |  |  |  |  |
| <20 | Reference |  | Reference |  | Reference |  |
| 20 – 50 | 2.041  (1.659 - 2.511) | <0.001 | 1.499  (1.365 - 1.647) | <0.001 | 0.963  (0.891 - 1.042) | 0.347 |
| >= 50 | 4.084  (3.286 - 5.076) | <0.001 | 2.615  (2.367 - 2.89) | <0.001 | 1.278  (1.171 - 1.394) | <0.001 |
| Abbreviations: LIS: Low-Income Status; COPD: Chronic Obstructive Pulmonary Disease; CCI: Deyo-Charlson Comorbidity Index; CNCP: Chronic Non-Cancer Pain; MME: Morphine Milligram. | | | | | | |

**Appendix 8:** **Sensitivity analysis 4**

**Methods:** A fourth sensitivity analysis was performed in which individuals who experienced OD, OUD, or all-cause mortality were defined as cases if they had at least 14 days of opioids within the baseline hazard period (H_0_ or 120 to 90 days prior to the date of the outcome). Similarly, controls were also required to have at least 14 days of opioids supply within the baseline hazard period.

**Results:** Among the full eligible cohort of 89,295, 1,213 cases for OD, 4,648 cases of OUD and 5,247 cases of mortality were identified. After controlling for patient socio-demographics and clinical characteristics, compared to older adults on a steady dose, the odds of experiencing OD were significantly lower for rapid tapering (aOR=0.62; 95%CI: 0.45-0.85) and higher for dose escalation (aOR=2.49; 95%CI: 1.90-3.26). Compared with steady dose, the odds of OUD were significantly lower for rapid tapering (aOR=0.69; 95%CI: 0.59-0.80) and higher for dose escalation (aOR=1.57; 95%CI: 1.39-1.78). Compared to steady dose, significantly higher odds for all-cause mortality were found among patients undergoing slow (aOR=1.17; 95%CI: 1.01-1.35), rapid tapering (aOR=1.45; 95%CI: 1.27-1.66), and dose escalation (aOR=1.61; 95%CI: 1.41-1.84). Changes in the study design to require individuals to have longer durations of opioid use during the baseline hazard period did not significantly impact study findings.

**Appendix Table 8**: Adjusted relationship between rate of opioid dose changes and OD, OUD, and all-cause mortality among patients on long-term opioid therapy with changed case and control definition

| Patient Characteristics | Overdose | | Opioid use disorder | | All-cause mortality | |
| --- | --- | --- | --- | --- | --- | --- |
|  | Adjusted Odds ratio (95% CI) | p-value | Adjusted Odds ratio (95% CI) | p-value | Adjusted Odds ratio (95% CI) | p-value |
| Rate of opioid dose changes |  |  |  |  |  |  |
| Steady dose | Reference |  | Reference |  | Reference |  |
| Slow Tapering | 1.017 (0.749 - 1.382) | 0.912 | 0.985 (0.860 - 1.129) | 0.832 | 1.167 (1.012 - 1.346) | 0.034 |
| Rapid tapering | 0.615 (0.446 - 0.847) | 0.003 | 0.686 (0.591 - 0.796) | <.0001 | 1.452 (1.272 – 1.658) | <.0001 |
| Escalation | 2.488 (1.900 - 3.257) | <0.001 | 1.573 (1.387 – 1.784) | <.0001 | 1.610 (1.408 – 1.841) | <.0001 |
| Gender |  |  |  |  |  |  |
| Female | 0.999 (0.835 - 1.195) | 0.987 | 1.089 (1.001 – 1.184) | 0.0469 | 0.745 (0.677 - 0.820) | <.0001 |
| Race and ethnicity |  |  |  |  |  |  |
| Non-Hispanic White | Reference |  | Reference |  | Reference |  |
| Black | 0.671 (0.501 – 0.899) | 0.007 | 0.933 (0.820 – 1.062) | 0.291 | 0.756 (0.656 – 0.871) | <.0001 |
| Hispanic | 1.022 (0.703 - 1.485) | 0.91 | 0.939 (0.795 – 1.108) | 0.456 | 0.642 (0.536 - 0.768) | <.0001 |
| Other racial groups | 0.914 (0.582 - 1.485) | 0.697 | 1.122 (0.902 – 1.395) | 0.302 | 0.759 (0.590 – 0.975) | 0.031 |
| LIS enrollment |  |  |  |  |  |  |
| Yes | 1.050 (0.887 - 1.243) | 0.572 | 1.030 (0.950 – 1.116) | 0.473 | 1.632 (1.500 - 1.777) | <0.001 |
| Multiple CNCP | 1.950 (1.647 - 2.309) | <0.001 | 1.679 (1.546 - 1.823) | <0.001 | 0.868 (0.798 – 0.945) | <0.001 |
| Renal impairment | 1.326 (1.042- 1.687) | 0.022 | 1.044 (0.929 - 1.172) | 0.471 | 2.154 (1.943 - 2.388) | <0.001 |
| Hepatic impairment | 1.053 (0.436 - 2.542) | 0.909 | 0.975 (0.731 – 1.301) | 0.864 | 1.776 (1.312 - 2.404) | <0.001 |
| Substance use | 1.551 (1.303 - 1.846) | <0.001 | 1.243 (1.143 - 1.351) | <0.001 | 1.795 (1.631 – 1.976) | <0.001 |
| Sleep disorders | 1.449 (1.194 - 1.758) | <0.001 | 1.025 (0.935 - 1.124) | 0.6 | 2.436 (2.213 - 2.681) | <0.001 |
| Parkinson | 0.915 (0.549 - 1.524) | 0.733 | 0.984 (0.761 - 1.273) | 0.904 | 1.844 (1.487 - 2.288) | <0.001 |
| Mental disorder | 1.718 (1.245 - 2.371) | 0.001 | 1.039 (0.918 - 1.175) | 0.545 | 1.486 (1.299 – 1.701) | <0.001 |
| COPD | 1.105 (0.801 - 1.525) | 0.543 | 0.96 6(0.851 – 1.096) | 0.587 | 1.325 (1.158 – 1.517) | <0.001 |
| Concomitant medication use | N/A | N/A | 1.080 (0.996 – 1.171) | 0.064 | 1.204 (1.108 – 1.310) | <0.001 |
| CCI |  |  |  |  |  |  |
| Zero | Reference |  | Reference |  | Reference |  |
| 1_2 | 1.113 (0.925 - 1.338) | 0.257 | 1.007 (0.921 - 1.101) | 0.878 | 1.667 (1.515 – 1.835) | <0.001 |
| >=3 | 1.207 (0.929 - 1.569) | 0.16 | 0.846 (0.742 – 0.965) | 0.013 | 3.293 (2.886 – 3.757) | <0.001 |
| Baseline Opioid mean daily dose (MME) |  |  |  |  |  |  |
| <20 | Reference |  | Reference |  | Reference |  |
| 20 – 50 | 1.906 (1.535 - 2.368) | <0.001 | 1.590 (1.436 – 1.759) | <0.001 | 0.989 (0.899 - 1.088) | 0.818 |
| >= 50 | 3.789 (2.968 - 4.837) | <0.001 | 2.655 (2.368 - 2.977) | <0.001 | 1.251 (1.115 - 1.403) | <0.001 |
| Abbreviations: LIS: Low-Income Status; COPD: Chronic Obstructive Pulmonary Disease; CCI: Deyo-Charlson Comorbidity Index; CNCP: Chronic Non-Cancer Pain; MME: Morphine Milligram. | | | | | | |
|  |  |  |  |  |  |  |
